# New Compounds with Enhanced Biological Activity Through the Strategic Introduction of Silylated Groups into Hydroxystearic Acids

**DOI:** 10.3390/molecules30030440

**Published:** 2025-01-21

**Authors:** Chiara Zalambani, Lorenzo Anconelli, Natalia Calonghi, Dario Telese, Gabriele Micheletti, Carla Boga, Giovanna Farruggia, Eleonora Pagnotta

**Affiliations:** 1 Department of Pharmacy and Biotechnology, University of Bologna, Via San Donato 15, 40127 Bologna, Italy; chiara.zalambani2@unibo.it (C.Z.); lorenzo.anconelli3@unibo.it (L.A.); giovanna.farruggia@unibo.it (G.F.); 2 Department of Industrial Chemistry ‘Toso Montanari’, Alma Mater Studiorum Università di Bologna, Via Piero Gobetti 85, 40129 Bologna, Italy; dariotelese@virgilio.it (D.T.); gabriele.micheletti3@unibo.it (G.M.); 3 Research Centre for Cereal and Industrial Crops (CREA-CI), CREA Council for Agricultural Research and Economics, Via di Corticella 133, 40128 Bologna, Italy; eleonora.pagnotta@crea.gov.it

**Keywords:** (*R*)-9-hydroxystearic acid, oleic acid, silyl derivatives, colon cancer, lipid metabolism

## Abstract

In the field of medicinal chemistry, the introduction of silylated groups is an important strategy to alter the activity, selectivity, and pharmacokinetics of compounds based on the diverse traits of silicon, including atomic size, electronegativity, and hydrophobicity. The hydroxy group on C-9 or C-9 and C-10 of hydroxystearic acids have been functionalized as t-butyl dimethyl silyl ether. The target compounds have been fully characterized and tested for in vitro cytotoxicity in tumor cells HT29, HCT116, CaCo2, HeLa, MCF7, U2OS, and Jurkat J6 and normal I407 cells. In particular, the silyl derivative of (*R*)-9-hydroxystearic acid was more active in colon cancer cells. Analyses of cell proliferation, oxidative cell status, histones post-translational modifications, protein phosphorylation, gene expression, and DNA damage were performed to obtain information on the antitumor properties of the new molecules in comparison with the unmodified (*R*)-9-hydroxystearic acid’s previously studied effects. Our results suggest that the incorporation of a silyl functionality may be a useful tool for the structural development of new pharmaceutically active compounds against colon cancer.

## 1. Introduction

Lipids are a heterogeneous class of compounds that include simple fatty acids as well as complex multi-chain structures. Lipids fulfill vital functions both as structural components of membranes and as energy reserves but above all as signaling molecules involved in the regulation of various metabolic and immune processes. Previous work has shown that 9-hydroxystearic acid (9-HSA) inhibits histone deacetylase 1, leading to cell cycle arrest in the G0/G1 phase and thereby promoting inhibition of cell growth and differentiation in many tumor cells. 9-Hydroxystearic acid, an endogenous lipid capable of inhibiting cell growth in a variety of tumor cell lines, has been well characterized for its biological activity in HT29, a colon carcinoma cell line. 9-HSA selectively inhibits the histone deacetylase HDAC1 and causes an arrest in the G0/G1 phase of the cell cycle in HT29 [1,2,3,4,5]. In addition, (*R*)-9-HSA has been investigated for potential biomedical applications by incorporating it into biocompatible keratin nanoparticles [6], and magnetic nanoparticles [7]. In recent years, the attention of many researchers has been focused on fatty acyl esters of hydroxy fatty acids (FAHFAs), which is a class of endogenous mammalian lipids that plays significant effects on metabolic control, acting as antidiabetic and anti-inflammatory agents [8,9]. In particular, of all derivatives, 5- and 9-PAHSA have significant antidiabetic effects, but only 9-PAHSA has anti-inflammatory activity, highlighting regiospecific biological selectivity [10]. The importance of the absolute configuration for the biochemistry and biological activity of lipids prompted the researchers to develop the stereoselective synthesis of PAHSA and then to identify (*R*)-9-PAHSA as the natural isomer in adipose tissue [11]. Although the (*R*)-enantiomer of 9-hydroxystearic acid is a good anticancer agent, its micromolar activity requires new solutions to improve its efficacy and bioavailability. Replacing carbon with silicon in biologically pre-validated compounds may change the shape, charge, and lipophilicity of the molecules, which in turn can positively influence both the biological activity and anticancer properties of the parent compounds. In particular, an increase in lipophilicity can significantly increase the distribution volume of the drug and thus improve its internalization into the tissue. An important observation is that lipophilic derivatives of anticancer drugs appear to be preferentially taken up by tumors, suggesting that an increase in lipophilicity may lead to an increase in drug concentration in tumor tissue [12,13,14,15]. These findings led us to further investigate the benefits of silyl functionality on hydroxy stearic acids. The hydroxy group at C-9 or C-9 and C-10 of hydroxystearic acids was functionalized as *t*-butyl dimethylsilyl ether. The novel products have been fully characterized and their biological activity assessed.

## 2. Results and Discussion

### 2.1. Synthesis of O-Silylated Hydroxystearic Acids

**Scheme 1:**
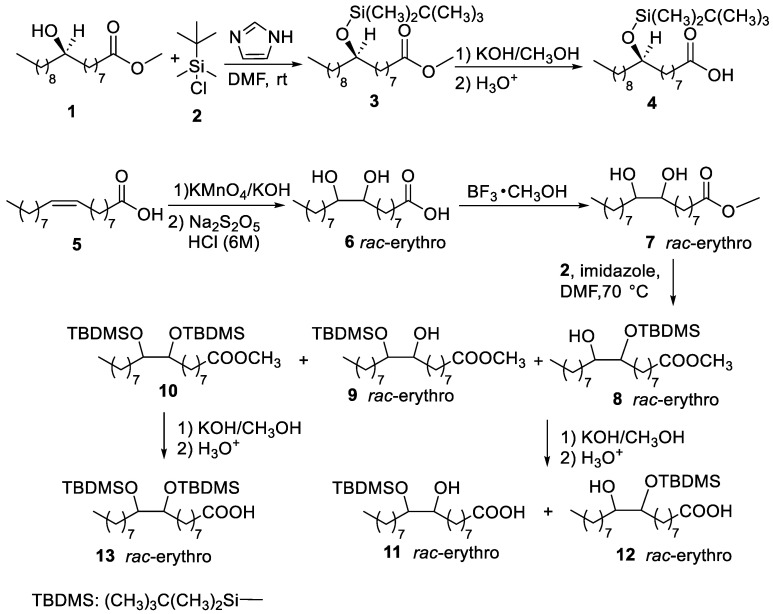
Synthetic routes to novel silylderivatives of (*R*)-9-hydroxystearic acid (**top**) and oleic acid (**bottom**).

Novel silyl derivatives of 9-hydroxystearic acid and of erythro-9,10-dihydroxystearic acid have been synthesized as shown in Scheme 1 (top). Methyl (*R*)-9-((tert-butyldimethylsilyl)oxy)octadecanoate (**3**) was obtained by a reaction between methyl-(*9R*)-9-hydroxystearate (**1**), obtained from *Dimorphoteca sinuata* seeds [5], and *tert*-butylchlorodimethylsilane (**2**), in *N*,*N*,-dimethylformamide (DMF) and in the presence of imidazole. Alkaline hydrolysis of **3** followed by acidification produced in good yield (*R*)-9-((tert-butyldimethylsilyl)oxy)octadecanoic acid (**4**). The silyl ethers **8**–**10** of 9,10-dihydroxystearic acid have been obtained from oleic acid (**5**) through the synthetic sequence shown in Scheme 1 (bottom). First, stereospecific cis-hydroxylation of oleic acid (**5**) with potassium permanganate in an alkaline medium by a classical procedure [16,17,18,19] produced *rac*-erythro-9,10-dihydroxystearic acid (**6**), which, after methylation to **7**, was reacted with **2** in DMF and in the presence of imidazole, thus producing mono- (**8** and **9**) and di-*O*-silylated (**10**) derivatives. Compound **10** was separated from the mixture with mono-derivatives (**8** + **9**) by column chromatography on silica gel, but the mono-silylated positional isomers **8** and **9** were obtained in the mixture and not separated. Compound **10** has also been obtained from the mono-silylated mixture (**8** + **9**) by a further silylation step. The alkaline hydrolysis of the methyl esters gave the corresponding acids (mixture **11** + **12**) and **13**. Compounds **8**–**13** have been characterized by IR, NMR, and mass data (see Section 3).

To the best of our knowledge, compounds **3**, **4**, **8**, **9**, and **11**–**13** have not been reported so far, while only partial mass data of **10** are known [20].

### 2.2. Biological Studies

#### 2.2.1. Cell Viability

The antiproliferative effects of 9-HSA in a series of human tumor cell lines (HT29, MCF-7, HeLa, U2OS, and J6) have been previously demonstrated. In these lines, 9-HSA showed biological activity in a range between 10 and 50 µM [4,21]. The in vitro anticancer activities of all compounds were evaluated using the MTT assay against the same series of human tumor cell lines. As shown in Table 1, compounds (**8** + **9**) and **10** showed no activity in any cell line in a concentration range of 10–500 nM. These compounds are 9-hydroxystearic acid methyl esters derivatized with silyl groups in positions C-10 and in C-9-C-10, respectively. In contrast, compounds (**11** + **12**), **13**, **3**, and **4** showed antitumor activity against all cell lines tested. All these compounds showed stronger antitumor activity in HT29 and U2OS cells. Compounds (**11** + **12**) are the acids bearing a silyl group at the 9-position, while **13** is silylated at both the 9- and 10-positions. Compounds (**11** + **12**) and compound **13** have higher activity in HT29 and U2OS, with an IC_50_ of 26.11 ± 0.43 nM and 25.32 ± 0.27 nM in HT29 and an IC_50_ of 50.40 ± 0.30 nM and 41.95 ± 0.29 nM in U2OS, respectively. Compound **3** is the (*R*)-9-enantiomer, which is methylated at the carboxyl group and silylated at the 9-position. This compound also shows higher activity in U2OS, with an IC_50_ of 29.33 ± 0.23 nM, compared to HT29, with 84.76 ± 0.10 nM. Compound **4** is the (*R*)-enantiomer of hydroxystearic acid silylated at the 9-position. Although this compound acts at nM concentrations in all tested lines, it shows greater activity in HT29 with an IC_50_ of 20.31 ± 0.24 nM, indicating selectivity against colon cancer.

**Figure 1:**
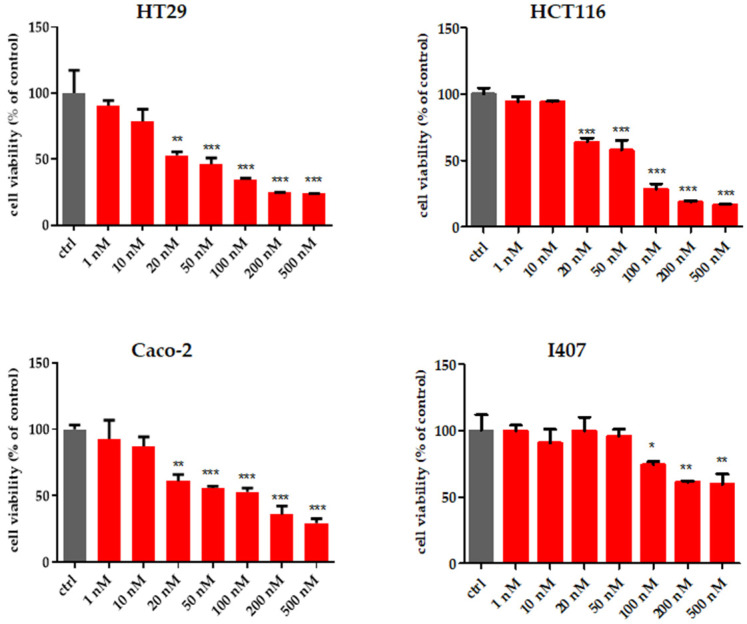
Compound **4** inhibits colon cancer cell growth in vitro. Cell viability of colon cancer HT29, HCT116, Caco-2, and normal I407 cells determined by MTT assays after 24 h of treatment with compound **4**. Data are represented as mean ± SD (n = 4). Significant differences against untreated controls (grey bars) are indicated as * *p* < 0.05, ** *p* < 0.005 and *** *p* < 0.0001.

For this reason, cytotoxicity was investigated in different colon cancer cell lines such as HCT-116 and CaCo-2 and in I407, a normal intestinal line. Cytotoxicity is shown in Figure 1. IC_50_ values and the Selective Index (S.I.) are reported in Table 2, indicating higher activity in all three tumor lines compared to the normal cell line, with a good selectivity index.

The effects of 9-HSA in HT29 have been deeply studied and reported in several papers [1,2,3,5]. For this reason, we focused our study on this cell line to compare the effect of the silylated derivative **4** to the free fatty hydroxylated acid.

#### 2.2.2. Effect on Cell Proliferation

**Figure 2:**
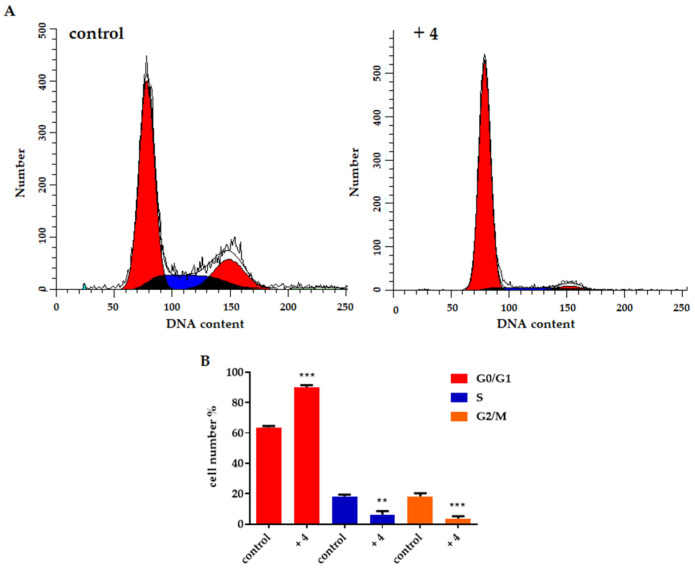
(**A**,**B**) The effect of **4** on the cell cycle of HT29 cells. HT29 cells were treated with 50 nM **4** for 24 h, fixed, and stained with propidium iodide (PI), and the cell cycle was analyzed by flow cytometry. Data are represented as mean ± SD. Significant differences are indicated as ** *p* < 0.01 and *** *p* < 0.001.

To better understand the process by which compound **4** increases cytotoxicity in the HT29 cell line, a cell cycle analysis was performed using flow cytometry. A dose of 50 nM was used for the assay. The experimental results are shown in Figure 2. They show a clear effect of the drug administered to HT29 cells and the observed increase in the G0/G1 phase by 41.8 ± 0.2%, followed by a decrease in S and G2/M phases by 66.4 ± 8.9% and 80.3 ± 6.1%, respectively. These results indicate that **4** induces cell cycle arrest in the G0/G1 phase and inhibits cell proliferation at 50 nM, while unmodified 9HSA or (*R*)-9HSA induced the same effect on the cell cycle at 100 µM or 50 µM, respectively [1,5].

Various mechanisms can be responsible for such a serious change in the cell cycle, and among them, reactive oxygen species (ROS) often play an important role. ROS have been implicated in cancer for a long time, and several types of cancer cells are known to have elevated levels of ROS compared to normal cells [22]. In general, increased ROS concentrations promote tumorigenicity by activating proliferative signaling pathways, impairing the function of tumor suppressor genes, increasing glucose and fatty acid metabolism, causing adaptations to hypoxia and triggering oncogenic mutations [23,24,25,26,27,28].

**Figure 3:**
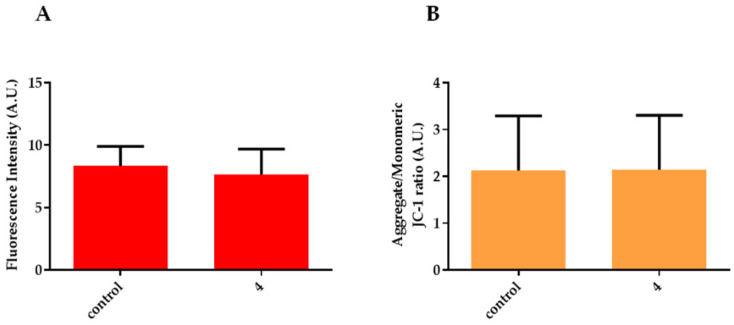
Effect of compound **4** on intracellular ROS level and mitochondrial potential. (**A**) Intracellular ROS content evaluated by DClF cellular fluorescence intensity, expressed as the fold increase with respect to the negative unstained cells. (**B**) The ratio between JC-1 red fluorescence and green fluorescence.

However, increasing ROS could trigger cell death, in particular, inducing apoptosis [29,30] and ferroptosis [31]. It is one of the mechanisms of action of several antitumor drugs [32,33]. For this reason, various molecules are used to eliminate or increase ROS levels and can be considered as potentially effective cancer therapies. Furthermore, antiproliferative molecules could impair mitochondrial function, both in ROS-dependent and -independent ways. Because of this, we tested whether the antiproliferative effect induced by compound **4** is related to changes in ROS levels or in mitochondrial potential. Interestingly, a 24 h treatment with compound **4** does not lead to a change in either the ROS content or the mitochondrial potential, as shown in Figure 3A,B.

**Figure 4:**
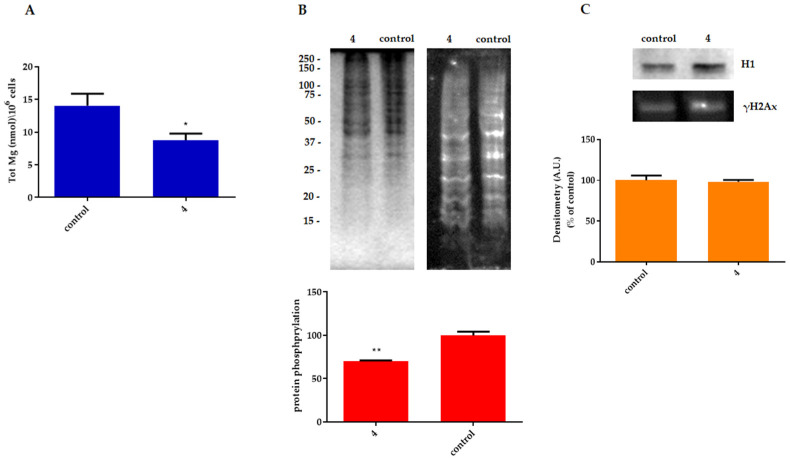
Effects of compound **4** on intracellular Mg content, phosphorylation of tyrosine residues, and DNA damage. (**A**) Total intracellular Mg as determined by the DCHQ5 assay (**B**) (**Top**) Western blotting analysis of tyrosine residue phosphorylation in HT29 control and treated with **4**. (**Left**) Representative image of HT29 cell lysate separated on SDS gel. (**Right**) Representative Western blot image. (**Bottom**) Quantification of protein expression as assessed by densitometry and normalized to total protein content. (**C**) Analysis of DNA damage. (**Top**) Representative Western blot image of γH2AX in control and HT29 cells treated with **4** (50 nM) for 6 h. (**Bottom**) Relative quantification. Arbitrary densitometry units (A.U.) were normalized by histone H1. All data represent the mean ± SD (n = 3). * *p* < 0.05, ** *p* < 0.001, compared to untreated control cells.

We then evaluated if compound **4** could affect the proliferative signal transduction. Receptor tyrosine kinases (RTKs) in their wild-type form and subsequent activation with their specific ligands are responsible for proliferation, survival, and migration mechanisms [34]. The activation of these receptors is involved in crucial signaling pathways and cell functions, processes that also depend on magnesium (Mg) [35]. Figure 4A shows how a 24 h treatment with **4** leads to a significant reduction in the Mg content (−40%). Interestingly, a decrease in phosphorylation levels at tyrosine residues of proteins is observed. As shown in Figure 4B, phosphorylation decreases most at tyrosine residues of proteins with a molecular weight above 50 KDa. In addition, Mg also plays an important role in the stabilization of DNA, and in particular, at low concentrations, it can lead to its destabilization [36,37]. Therefore, we analyzed the phosphorylation state of histone H2AX after 6 h of treatment with **4**. As shown in Figure 4C, **4** does not induce any changes in the γH2AX marker, suggesting that the significant reduction in Mg levels is not associated with DNA damage. These observations, therefore, indicate that the decrease in Mg alone can trigger a cell cycle arrest in the G0/G1 phase and are in agreement with the data available in the literature [38].

#### 2.2.3. Compound **4** Treatment Reduced the Intracellular Lipid Accumulation

**Figure 5:**
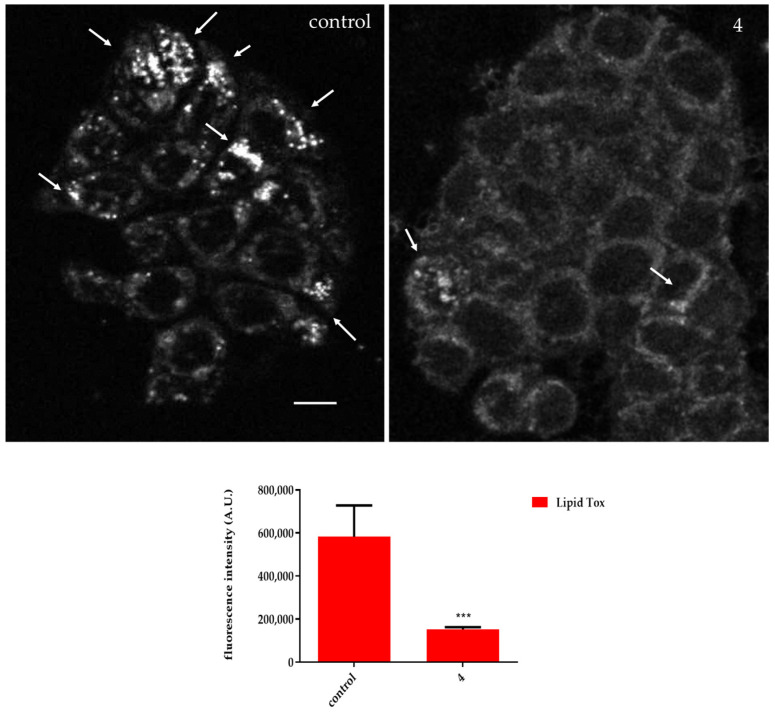
Neutral lipid staining in HT29. (**Top**) Representative images of HT29 untreated control cells and treated with the compound **4** (50 nM) for 48 h. Cellular lipids were stained with LT, and cells were observed under a confocal fluorescence microscope. White arrows indicate lipid droplets. (**Bottom**) The total intensity (number of pixels × mean intensity) of the LT spot signal was quantified using ImageJ software (https://imagej.net/software/imagej/; access date 25 October 2024). Results are shown as mean fluorescence intensity ± SD and were obtained from three independent experiments with at least 5 images per sample. Scale bar: 20 µm. Significant difference is indicated as *** *p* < 0.001, compared to untreated control cells.

**Figure 6:**
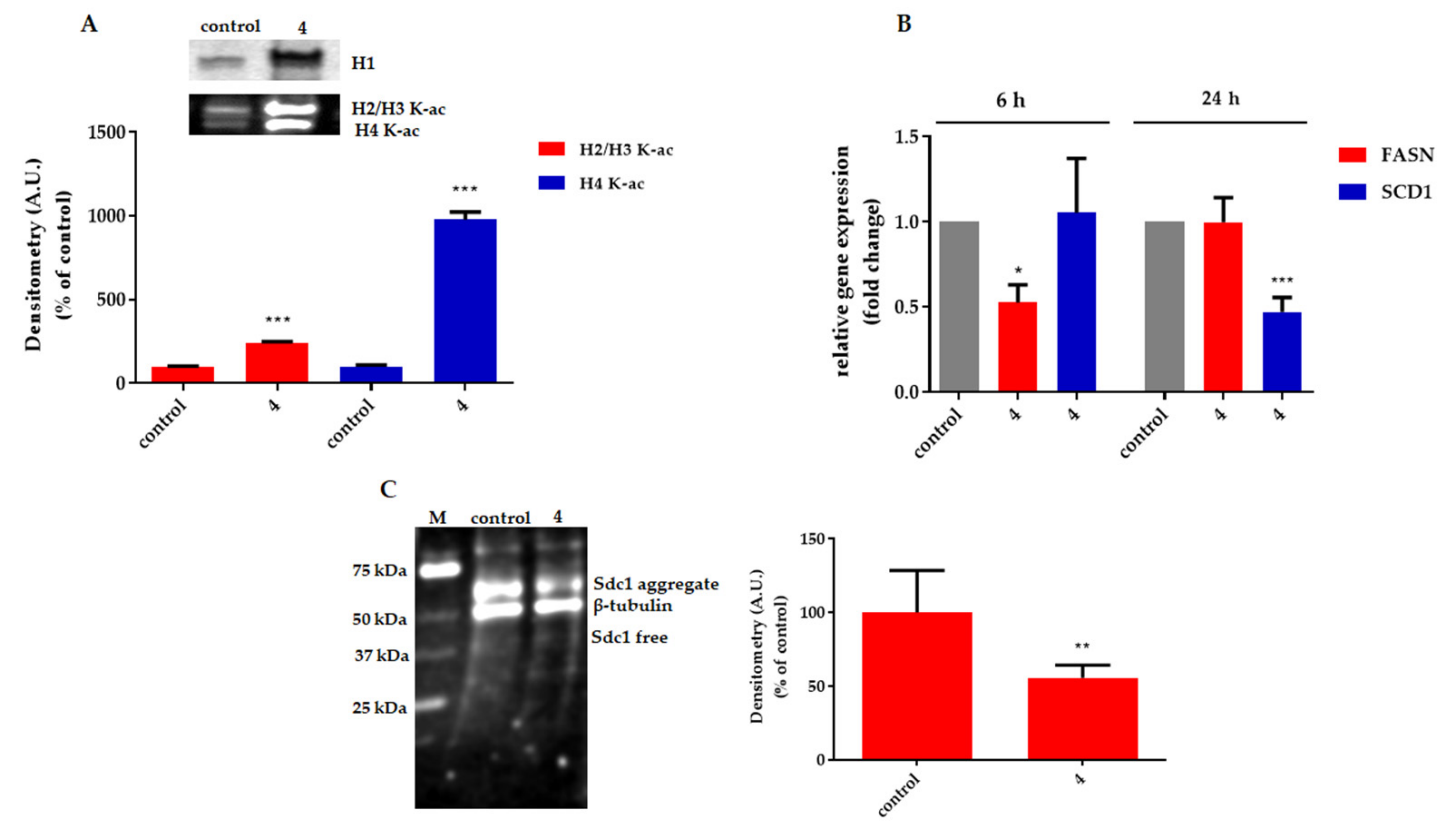
Compound **4** alters histone acetylation status as well as gene and protein expression. (**A**) The acetylation status of histones H2/H3 and H4 of HT29 cells treated with **4** (50 nM) for 6 h. (**Top**) Representative Western blotting images of acetylated histones. (**Bottom**) Relative quantification of H2/H3 and H4 acetylation. Arbitrary densitometry units (A.U.) were normalized by histone H1. (**B**) Normalized gene expression for FASN and SCD1 analyzed by RT-PCR. Data are expressed as means of relative expression (fold change) between treatment **4** and the untreated control sample (grey bars). (**C**) (**Left**) Representative Western blot image of scd1 in HT29 cells treated with **4** (50 nM) for 48 h. (**Right**) Relative quantification of the protein. All data are mean ± SD (n = 3). Significant differences are indicated by * *p* ≤ 0.05, ** *p* ≤ 0.005, and *** *p* < 0.001, compared to untreated control samples.

In cells, a reduction in the proliferation rate is also accompanied by a change in metabolic activity so that the cell can adapt to a new state of life. In colon cancer, in particular, the changes in metabolism lead to impaired production of various lipids, which serve as energy sources to meet the need for rapid proliferation. Long-chain fatty acids are important for the synthesis of membranes in proliferating cells and also play a key role in various signaling pathways [39]. For their synthesis as components of cell membranes, the relationship between the length of the chain and the degree of saturation is important, as they influence both the maintenance of fluidity and the curvature of the membrane [40]. Tumor cells often overexpress key enzymes responsible for the de novo biosynthesis of fatty acids, such as acetyl-CoA carboxylase (ACACA), ATP citrate lyase (ACLY), and fatty acid synthase (FASN), whose cascade activity leads to the synthesis of palmitic acid, with increased expression of FASN found in almost all human tumor types [41,42,43,44,45,46,47,48,49]. Therefore, FASN is considered a metabolic oncogene. Following the synthesis of palmitate, a crucial step is the production of monounsaturated fatty acids (MUFAs) by the activation of stearoyl-CoA desaturase (SCD), which together with various elongases (ELOVL) produces a broad spectrum of fatty acids (FAs) in cell membranes [50]. SCD plays an important role in controlling tumor cell metabolism, as its inhibition leads to a blockade of tumor cell proliferation, reduces their survival, and restricts the ability of tumor stem cells to initiate tumors [51,52,53]. Furthermore, SCD1 converts saturated fatty acids (SFAs) into monounsaturated fatty acids (MUFAs), thus promoting colorectal cancer (CRC) metastasis [54]. In conclusion, SCD1 is an interesting new target for the development of novel therapeutic agents for the treatment of CRC metastasis. To determine whether compound **4** can alter lipid metabolism, we performed a confocal microscopy assay by labeling the cells with LipidTOX™(LT). Figure 5 shows how a 48 h treatment with compound **4** significantly reduces the accumulation of lipid droplets, suggesting that this compound not only suppresses carcinoma proliferation but also reduces the accumulation of neutral lipids, indicating an important alteration in lipid metabolism. After observing the decrease in the lipid component in HT29 treated with **4**, we examined the changes in the acetylation state of histone proteins and the temporal spectrum of FASN and SCD1 mRNA expression. Interestingly, there is instead an extended increase in the acetylation state of histones H2/H3 and H4, in comparison to unmodified 9HSAs [2,5], suggesting that treatment with **4** leads to different epigenetic modifications, with a consequent change in gene transcription, as shown in Figure 6A.

The acetylation and deacetylation of lysine residues in histone proteins play a crucial role in the control of gene transcription. These functions are controlled by two enzyme activities, acetyltransferases (HATs) and deacetylases (HDACs), which act as coregulators in DNA transcription, replication, and repair [55]. The mechanisms by which acetylation and deacetylation control transcription involve two main pathways. First, histone acetylation opens chromatin structure, resulting in DNA accessibility in specific regions. Second, lysine residues and their modifications in the form of acetylation and deacetylation provide specific binding sites for repressors and activators of gene activity [56].

The changes in histone acetylation are accompanied by a change in gene expression characterized by a marked down-regulation of the FASN gene after 6 h of treatment, while SCD1 expression decreases after 24 h (Figure 6B). The decrease in SCD1 gene expression leads to a decrease in the amount of its protein product, as can be clearly seen in Figure 6C. In particular, the anti-scd1 antibody recognizes the protein both in its free form between 25 and 37 kDa and in the aggregated form with a MW of about 70 kDa. This is probably due to the denaturation condition used (100 °C for 2 min) that is required for the electrophoretic protocol, which uses MOPS running buffer.

## 3. Materials and Methods

### 3.1. Chemical Synthesis

#### 3.1.1. General

The nuclear magnetic resonance spectra (1H NMR and 13C NMR) were recorded at 25 °C on Varian spectrometers Gemini 300, Mercury 400, or Inova 600 (Varian, Palo Alto, CA, USA) operating at 100, 400, or 600 MHz (for ^1^H-NMR) and 75, 100.56, or 150.80 MHz (for ^13^C-NMR), respectively. CHn multiplicity was established by DEPT-135 experiments. Chemical shifts were measured in δ (ppm) with reference to the solvent (CDCl3: δ = 7.26 ppm for ^1^H NMR and δ = 77.0 ppm for ^13^C NMR). *J* values are given in Hz. Electrospray ionization (ESI)-MS spectra and ESI high-resolution HRMS spectra were recorded using a Waters ZQ 4000 (Waters Corporation, Milford, MA, USA) and a Xevo (Waters Corporation, Milford, MA, USA) instrument, respectively. IR spectra were recorded using a Fourier transform spectrophotometer Spectrum Two in the 4000–400 cm^−1^ wavelength range, using a Universal ATR accessory (Perkin Elmer, Waltham, MA, USA). For flash chromatography (FC), silica gel 0.037–0.063 mm (Merck KGaA, Darmstadt, Germany) was used as the stationary phase. Thin-layer chromatography (TLC) was carried out on silica gel 60 (Fluka Analytical, Buchs, Switzerland), and the spots were revealed using an aqueous solution of (NH_4_)_6_MoO_24_ (2.5%) and (NH_4_)_4_Ce(SO_4_)_4_ (4%) in 10% H_2_SO_4_. The yields of reactions herein reported have not been optimized. Methyl-(*9R*)-9-hydroxystearate (**1**) was prepared according to the procedure previously reported by us by the authors of [5], and their chemico-physical data agree with those reported in [57]. Oleic acid (**5**) and all reagents and solvents used were purchased by Sigma-Aldrich (Milan, Italy).

#### 3.1.2. Synthesis of Methyl (*R*)-9-((*tert*-butyldimethylsilyl)oxy)octadecanoate (**3**)

To a solution of methyl (*9R*)-9-hydroxystearate (**1**, 0.100 g, 0.32 mmol) in *N*,*N*-dimethylformamide (5 mL) was added imidazole (0.052 g, 0.76 mmol) and *tert*-butyldimethylchlorosilane (0.082 g, 0.54 mmol). The reaction mixture was stirred at room temperature for 24 h, diluted with diethyl ether (20 mL), washed with H_2_O (2 × 10 mL) and brine (20 mL), dried over anhydrous MgSO_4_, and concentrated under reduced pressure. The residue was purified by flash silica gel column chromatography (light petroleum:diethyl ether 7:3 *v*/*v* ratio) to afford compound **3** (0.104 g, 76%) as a colorless oil.

^1^H-NMR (300 MHz, CDCl_3_, 25 °C) δ, ppm = 3.65 (s, 3H, OCH_3_), 3.60 (m, 1H, CHOSi), 2.28 (t, *J* = 7.5 Hz, 2H, CH_2_CO), 1.69–1.52 (m, 2H), 1.48–1.14 (m, 28H), 0.96–0.78 (s + t overlapped, 12H, (CH_3_)_3_Si + CH_3_), 0.02 (s, 6H, (CH_3_)_2_Si); ^13^C-NMR (75 MHz CDCl_3_, 25 °C) δ, ppm = 174.3 (C), 72.3 (CH), 51.4 (CH_3_), 37.13 (CH_2_), 37.07 (CH_2_), 34.1 (CH_2_), 31.9 (CH_2_), 29.8 (CH_2_), 29.65 (CH_2_), 29.58 (CH_2_), 29.3 (CH_2_), 29.2 (CH_2_), 29.1 (two signals overlapped, CH_2_), 25.9 (CH_3_), 25.3 (CH_2_), 25.2 (CH_2_), 24.9 (CH_2_), 22.7 (CH_2_), 18.1 (C) 14.1 (CH_3_), −4.4 (CH_3_). ESI-MS (*m*/*z*): 451 [M + Na]^+^, 467 [M + K]^+^. ATR-IR: (cm^−1^): 2927, 2855, 1744, 1463, 1436, 1361, 1252, 1196, 1170, 1058, 1006, 834, 808, 772, 722; HRMS (ESI^+^) calcd for C_25_H_52_NaO_3_Si (M + Na)^+^: 451.3583, found: 451.3600.

#### 3.1.3. Synthesis of (*R*)-9-((*tert*-butyldimethylsilyl)oxy)octadecanoic Acid (**4**)

Compound **3** (0.104 g, 0.24 mmol) was dissolved in 5 mL of (10% *w*/*v*) KOH in methanol and stirred for 2 h at room temperature. The reaction was monitored by TLC (eluent: petroleum ether/diethyl ether 7/3 *v*/*v*). The solvent was removed under vacuum, and the solid was dissolved in water and acidified with 6 M HCl until the acid precipitated as a white solid. This mixture was extracted with ethyl acetate (3 × 20 mL), and the combined organic layers were dried over anhydrous MgSO_4_. After filtration and solvent removal under reduced pressure, compound **4** was recovered as a yellowish oil (0.090 g, 0.22 mmol, 92% yield).

^1^H-NMR (300 MHz, CDCl_3_, 25 °C) δ, ppm = 3.60 (m, 1H, CHOSi), 2.34 (t, *J* = 7.6 Hz, 2H, CH_2_CO), 1.7–1.53 (m, 2H), 1.48–1.14 (m, 28H), 0.96–0.78 (s + t overlapped, (CH_3_)_3_Si + CH_3_, 12H), 0.03 (s, 6H, (CH_3_)_2_Si); ^13^C-NMR (75 MHz CDCl_3_, 25 °C) δ, ppm = 180.3 (C), 72.4 (CH), 37.13 (CH_2_), 37.10 (CH_2_), 34.1 (CH_2_), 31.9 (CH_2_), 29.8 (CH_2_), 29.7 (CH_2_), 29.64 (CH_2_), 29.58 (CH_2_), 29.3 (CH_2_), 29.2 (CH_2_), 29.0 (CH_2_), 25.9 (CH_3_), 25.3 (CH_2_), 25.2 (CH_2_), 24.6 (CH_2_), 22.7 (CH_2_), 18.1 (C), 14.1 (CH_3_), −4.4 (CH_3_). ESI-MS (*m*/*z*): 437 [M + Na]^+^. ATR-IR: (cm^−1^): 2926, 2855, 1710, 1463, 1253, 1068, 1005, 938, 834, 772, 722; HRMS (ESI^+^) calcd for C_24_H_50_NaO_3_Si (M + Na)^+^: 437.3427, found: 437.3431.

#### 3.1.4. Synthesis of *Rac*-erythro-9,10-dihydroxystearic Acid (**6**)

Oleic acid (5, 2.0 g, 7.0 mmol) was added to an aqueous solution of KOH (2.0 g, 36 mmol, 100 mL). The mixture was heated at 50 °C then cooled down to room temperature. The flask was placed in an external ice bath, and an aqueous solution of KMnO_4_ (2.1 g in 150 mL of water) was added dropwise at 0 °C. After 10 min, sodium thiosulfate was added, and then the mixture was acidified with 37% HCl solution and filtered. The white solid obtained was recrystallized from methanol, and 9,10-dihydroxystearic acid (**6**) was recovered as a white solid (1.61 g, 73%), mp: 128–130 °C (Lit.: 121–130 °C); other chemico-physical data agree with those reported in the literature [58].

#### 3.1.5. Synthesis of *Rac*-erythro-methyl 9,10-dihydroxystearate (**7**)

Compound **6** (1.57 g, 5.0 mmol) and 15 mL of 14% BF_3_/MeOH solution were placed in a high-pressure vessel and sealed. The system was kept under magnetic stirring and heated at 70 °C. After 2 h, the methylation was completed by TLC (eluent: *n*-hexane:ethyl acetate 1:5 *v*/*v*). The mixture was allowed to stand at room temperature; then, it was treated with brine (10 mL) and 6 M HCl and extracted with dichloromethane (3 × 30 mL). The combined organic layer was dried over MgSO_4_ and filtered. After removal of the solvent, a white solid was recovered, which was dissolved in methanol and treated again with aq. HCl until the precipitation of a white solid. After filtration over a Buchner funnel, washing with cold methanol (0 °C), and drying in a desiccator, compound **7** (1.43 g, 86%) was obtained, and the chemico-physical data agree with those reported in [59].

#### 3.1.6. Synthesis of *Rac*-erythro-methyl 9-((*tert*-butyldimethylsilyl)oxy)-10-hydroxyoctadecanoate and *Rac*-erythro-methyl 10-((*tert*-butyldimethylsilyl)oxy)-9-hydroxyoctadecanoate (**8** and **9**)

In a three-necked round-bottomed flask, dried and under a nitrogen atmosphere, compound **7** (0.208 g, 0.63 mmol) was dissolved in *N*,*N*-dimethylformamide (8 mL). Imidazole (0.107 g, 1.58 mmol) and *tert*-butyldimethylchlorosilane (0.476 g, 3.15 mmol) were consecutively added. The reaction mixture was stirred at 100 °C overnight. TLC analysis (*n*-hexane:ethyl acetate 5:1 *v*/*v*) revealed an incomplete conversion of starting **7**. To the mixture, a further amount of imidazole (0.107 g, 1.58 mmol) and *tert*-butyldimethylchlorosilane (0.476 g, 3.15 mmol) was added. After 1 h, the conversion was complete. To the mixture of diethyl ether (10 mL), 5% aq. HCl was added, and after extraction with Et_2_O (3 × 10 mL), the collected organic layers were washed with aq. NaHCO_3_, H_2_O (2 × 10 mL), and brine (20 mL). The organic layer was dried over anhydrous MgSO_4_, filtered, and concentrated under reduced pressure. The residue was purified by flash silica gel column chromatography (light petroleum:diethyl ether 9:1 *v*/*v*) to afford a trace of compound **10** and the isomeric mixture **8** + **9** (0.121 g, 43%) as a colorless oil. ^1^H-NMR (600 MHz, CDCl_3_, 25 °C) δ, ppm = 3.66 (s, 3H, OCH_3_), 3.62-3.52 (m, 2H, CHOSi + CHOH), 2.30 (t, *J* = 7.6 Hz, 2H, CH_2_CO), 1.67–1.55 (m, 2H), 1.54–1.15 (m, 24H), 0.93–0.83 (m, 12H, (CH_3_)_3_Si + CH_3_), 0.07 (s, 6H, (CH_3_)_2_Si); ^13^C-NMR (150 MHz CDCl_3_, 25 °C) δ, ppm = 174.28 (C), 174.27 (C), 75.34 (CH), 75.29 (CH), 74.64 (CH), 74.62 (CH), 51.42 (two signals overl., CH_3_), 34.07 (two signals overl., CH_2_), 31.86 (two signals overl., CH_2_), 31.84 (CH_2_), 31.79 (CH_2_), 31.72 (two signals overl., CH_2_), 30.53 (CH_2_), 30.44 (CH_2_), 29.83 (CH_2_), 29.76 (CH_2_), 29.64 (two signals overl., CH_2_), 29.55 (CH_2_), 29.53 (CH_2_), 29.52 (CH_2_), 29.25 (CH_2_), 29.17 (CH_2_), 29.09 (CH_2_), 29.07 (CH_2_), 26.18 (CH_3_), 26.14 (CH_3_), 25.86 (CH_2_), 25.73 (CH_2_), 25.68 (CH_2_), 24.91 (two signals overl., CH_2_), 22.65 (CH_2_), 18.06 (two signals overl., C), 14.08 (CH_3_), 14.07 (CH_3_), −4.44 (two signals overl., CH_3_). ATR-IR: (cm^−1^): 3510, 2951, 2925, 2856, 1742 (C=O), 1462, 1436, 1360, 1252, 1078, 834, 775. ESI-MS (*m*/*z*): 467 [M + Na]^+^, 483 [M + K]^+^; HRMS (ESI^+^) calcd for C_25_H_52_NaO_4_Si (M + Na)^+^: 467.3533, found: 467.3546.

#### 3.1.7. Synthesis of *Rac*-erythro-methyl 9,10-bis((*tert*-butyldimethylsilyl)oxy)octadecanoate (**10**)

The reaction was carried out starting from **8** + **9** in the same manner described for the preparation of the monosilylated oleic derivatives **8** + **9**. After purification by FC, pure compound **10** was recovered in 36% yield as a colorless oil.

^1^H-NMR (600 MHz, CDCl_3_, 25 °C) δ, ppm = 3.66 (s, 3H, OCH_3_), 3.57-3.52 (m, 2H, CHOSi), 2.30 (t, *J* = 7.7 Hz, 2H, CH_2_CO), 1.67–1.57 (m, 2H), 1.52–1.19 (m, 24H), 0.92–0.84 (s + t overlapped, 21H, ((CH_3_)_3_Si)_2_ + CH_3_), 0.05 (br.s, 6H, (CH_3_)_2_Si), 0.038 (s, 3H, CH_3_Si), 0.034 (s, 3H, CH_3_Si); ^13^C-NMR (150 MHz CDCl_3_, 25 °C) δ, ppm = 174.3 (C), 75.98 (CH), 75.93 (CH), 51.42 (CH_3_), 34.1 (CH_2_), 33.2 (CH_2_), 33.0 (CH_2_), 31.9 (CH_2_), 29.9 (CH_2_), 29.7 (CH_2_), 29.6 (CH_2_), 29.3 (CH_2_), 29.2 (CH_2_), 29.1 (CH_2_), 26.0 (two signals overl., CH_3_), 25.4 (CH_2_), 25.3 (CH_2_), 24.9 (CH_2_), 22.7 (CH_2_), 18.2 (two signals overl., C), 14.1 (CH_3_), −4.1 (CH_3_), −4.5 (CH_3_). ATR-IR: (cm^−1^): 3582, 2951, 2929, 2855, 1743 (C=O), 1463, 1380, 1357, 1252, 1096, 835. ESI-MS (*m*/*z*): 581 [M + Na]^+^, 597 [M + K]^+^; HRMS (ESI^+^) calcd for C_31_H_66_NaO_4_Si_2_ (M + Na)^+^: 581.4397, found: 581.4420.

#### 3.1.8. Synthesis of *Rac*-erythro-9-((*tert*-butyldimethylsilyl)oxy)-10-hydroxyoctadecanoic acid and *Rac*-erythro-10-((*tert*-butyldimethylsilyl)oxy)-9-hydroxyoctadecanoic Acid (**11** and **12**)

The mixture of monosilylated esters **8** + **9** (0.460 g, 1.04 mmol) was dissolved in 20 mL of a 10% (*w*/*v*) methanolic solution of KOH and stirred for 6 h at room temperature. The reaction was monitored by TLC (eluent: petroleum ether/diethyl ether 7/3 *v*/*v*). After the addition of water (40 mL) and acidification with aq. 37% HCl, brine (10 mL) was added, and the mixture was extracted with ethyl acetate (2 × 50 mL). The combined organic layers were dried over anhydrous MgSO_4_. After filtration and solvent removal under reduced pressure, the mixture of pure **11** + **12** was recovered as a yellow oil (0.277 g, 64 mmol, 62% yield).

^1^H-NMR (600 MHz, CDCl_3_, 25 °C) δ, ppm = 3.62–3.54 (m, 2H, CHOSi + CHOH), 2.34 (t, *J* = 7.6 Hz, 2H, CH_2_CO), 1.64 (q, *J* = 7.3, 2H), 1.54–1.16 (m, 24H), 0.94–0.84 (s + t overl., 12H, (CH_3_)_3_Si + CH_3_), 0.07 (s, 6H, (CH_3_)_2_Si); ^13^C-NMR (150 MHz CDCl_3_, 25 °C) δ, ppm = 179.2 (C), 75.33 (CH), 75.28 (CH), 74.7 (CH), 74.6 (CH), 33.9 (CH_2_), 31.87 (CH_2_), 31.84 (CH_2_), 31.77 (CH_2_), 31.68 (two signals overl., CH_2_), 30.5 (CH_2_), 30.4 (CH_2_), 29.8 (CH_2_), 29.7 (CH_2_), 29.6 (CH_2_), 29.5 (CH_2_), 29.2 (CH_2_), 29.1 (CH_2_), 28.9 (two signals overl., CH_2_), 26.16 (CH_3_), 26.12 (CH_2_), 25.8 (CH_2_), 25.7 (CH_2_), 25.6 (CH_2_), 24.64 (CH_2_), 24.63 (CH_2_), 22.6 (two signals overl., CH_2_), 18.1 (C), 14.08 (CH_3_), 14.07 (CH_3_), −4.4 (two signals overl., CH_3_). ATR-IR: (cm^−1^): 3582, 3418, 3181, 2930, 2857, 1713 (C=O), 1466, 1253, 1082, 838, 775. ESI-MS (*m*/*z*): 453 [M + Na]^+^, 469 [M + K]^+^; HRMS (ESI^−^) calcd for C_24_H_49_O_4_Si^−^ (M − H)^−^: 429.3406, found: 429.3398.

#### 3.1.9. Synthesis of *Rac*-erythro-9,10-bis((*tert*-butyldimethylsilyl)oxy)octadecanoic Acid (**13**)

The reaction was carried out starting from **10** in the same manner described for the preparation of **11** + **12**. Pure compound **13** was recovered in 50% yield as a colorless oil.

^1^H-NMR (400 MHz, CDCl_3_, 25 °C) δ, ppm = 3.58–3.51 (m, 2H, CHOSi), 2.34 (t, *J* = 7.9, 2H, CH_2_CO), 1.69–1.57 (q, *J* = 7.6, 2H), 1.54–1.16 (m, 24H), 0.92–0.84 (s + t overl., 21H, ((CH_3_)_3_Si)_2_ + CH_3_), 0.05 (br.s, 6H, (CH_3_)_2_Si), 0.04 (br.s, 6H, (CH_3_)_2_Si); ^13^C-NMR (100 MHz CDCl_3_, 25 °C) δ, ppm = 180.3 (C), 75.98 (CH), 75.93 (CH), 34.1 (CH_2_), 33.2 (CH_2_), 33.1 (CH_2_), 31.9 (CH_2_), 29.9 (CH_2_), 29.7 (CH_2_), 29.6 (CH_2_), 29.3 (CH_2_), 29.2 (CH_2_), 29.0 (CH_2_), 26.0 (two signals overl., CH_3_), 25.4 (CH_2_), 25.3 (CH_2_), 24.6 (CH_2_), 22.7 (CH_2_), 18.2 (two signals overl., C), 14.1 (CH_3_), −4.1 (CH_3_), −4.5 (CH_3_). ATR-IR: (cm^−1^): 3582, 2926, 2857, 1710 (C=O), 1473, 1462, 1253, 1096, 835, 775. ESI-MS (*m*/*z*): 567 [M + Na]^+^, 583 [M + K]^+^; HRMS (ESI^+^) calcd for C_30_H_64_NaO_4_Si_2_ (M + Na)^+^: 567.4241, found: 567.4265.

### 3.2. Biology

#### 3.2.1. Cell Culture and Treatments

The human colorectal adenocarcinoma (Caco-2), human colorectal adenocarcinoma (HT29), normal human intestinal (I407), human cervical cancer (HeLa), human breast cancer (MCF7), human bone osteosarcoma (U2OS), and human acute T-cell leukemia (Jurkat J6) cell lines were purchased from American Type Culture Collection (ATCC, Manassas, VA, USA). Cells were cultured in RPMI 1640 medium (Labtek Eurobio, Milan, Italy), supplemented with 10% FCS (Euroclone, Milano, Italy) and 2 mM L-glutamine (Sigma-Aldrich, Milano, Italy), at 37 °C and in a 5% CO_2_ atmosphere. The compounds were dissolved in DMSO in a 30–40 mM stock solution. In cell treatments, the final DMSO concentration never exceeded 0.1%.

#### 3.2.2. MTT Assay

Cells were seeded in a 96-well culture plastic plate (Sarsted, Milan, Italy), at 5 × 10^4^ cells/well and, after 24 h, were exposed to increasing concentrations of the different compounds (from 1 nM to 500 nM) dissolved in RPMI 1640 medium. An MTT assay was performed according to Micheletti et al. [60].

The absorbance at 570 nm was measured using a multi-well plate reader (Tecan, Männedorf, Switzerland), and data were analyzed by Prism GraphPad 8 software and expressed as IC_50_ nM.

#### 3.2.3. Cell Cycle Analysis

HT29 cells were treated with compound **4** for 24 h and then detached with 0.11% trypsin/0.02% ethylenediaminetetraacetic acid (EDTA) (Sigma-Aldrich, St. Louis, MO, USA), washed in PBS, and centrifuged. Cells were stained and analyzed according to Micheletti et al. [60].

#### 3.2.4. ROS Assay

ROS were detected by DClFDA staining. This probe is not fluorescent and is permeable to alive cells, where the acetometoxyesther is cleaved by the ATP-dependent intracellular estherases. This product is made fluorescent by the concomitant action of ROS, which peroxidize the molecule. The intensity of the fluorescence is, therefore, proportional to the production of intracellular ROS. HT29 cells were grown on a 96-well black plate, treated for 24 h with **4**, washed in Hanks’ buffer, stained with 2 µM DClFDA, and incubated at 37 °C in the dark for 30 min. Negative controls were treated in the same way, but DClFDA was not added to these samples. The cells were washed in fresh Hanks’ buffer and resuspended in Hanks’ buffer containing Hoechst 33342 1 µg/mL to stain cell nuclei, to evaluate the cell density in the wells. The fluorescences were read with the plate reader Ensight (Perkin Elmer, Waltham, MA, USA) with excitation wavelengths, respectively, 488 nm for DClF and 360 nm for Hoechst and emission wavelength 535 nm for DClF and 454 for Hoechst 33342. The intensity of DClF fluorescence was normalized on Hoechst fluorescence and is expressed in Arbitrary Units (AU).

#### 3.2.5. Analysis of Mitochondrial Potential by JC-1 Staining

JC-1 is a dye that is permeable to alive cells and is accumulated in the anionic mitochondrial matrix indicating the level of mitochondrial potential. When present at low concentrations, the JC-1 monomer fluorescence is green (around 535 nm), while at increased concentration, it can aggregate and fluoresces in the red (600 nm). The ratio between the red fluorescence and the green one is directly proportional to the mitochondrial activity. The HT29 cells were grown on a 96-well black plate, treated for 24 h with **4**, washed in Hanks’ buffer, stained with 2 µM JC-1, and incubated 30 min in the dark at 37 °C. Negative controls were treated in the same way, but JC-1 was not added to these samples. The plates were read on the Ensight plate reader, exciting the red fluorescence at 506 nm and the green one at 488 nm, while the emission was, respectively, read at 595 nm and 535 nm, and the ratio between the red and green fluorescence was calculated.

#### 3.2.6. Cellular Content of Total Magnesium

To evaluate the total magnesium, the fluorimetric DCHQ5 assay was performed according to Nature Protocol [61]. Briefly, the cells were washed in PBS without Mg and Ca three times, accurately counted, and sonicated at 100 W. A standard curve was prepared with a concentration of MgCl_2_ between 0.5 and 5 µM in buffer MOPS 2 mM in 50% MetOH. The fluorescent probe DCHQ5 15 µM was added, and the fluorescence was recorded on a PTI Quanta Master C60/2000 spectrofluorometer (Photon Technology International, Inc., Birmingham, NJ, USA). A total of 200 µL of the samples was diluted in the MOPS MetOH buffer, DCHQ5 15 µM was added, and the fluorescence was recorded. The amount of Mg was evaluated by interpolating the fluorescence of the sample with respect to the standard curve expressed as nmoles of magnesium/10^6^ cells.

#### 3.2.7. Histone Post-Translational Modification

HT29 were seeded at a density of 1.5 × 10^4^ cell/cm^2^ in a Petri dish and, after 24 h, treated for 6 h with 50 nM of compound **4**. Cells were harvested, and histones were extracted by previously isolated nuclei according to Micheletti et al. [60]. The proteins were resolved on a 10% gel in MES buffer at 200 V for 35 min and immunoblotted with mouse anti-acetylated lysines (Millipore, Billerica, MA, USA) or rabbit anti-γH2Ax (Santa Cruz Biotechnology, Dallas, TX, USA) primary antibodies. The membranes were developed with Clarity Western ECL Substrate (Bio-Rad, Hercules, CA, USA) after incubation with mouse or rabbit secondary horseradish peroxidase-conjugated antibody (GE Healthcare, Chicago, IL, USA). SDS-PAGE was stained with Coomassie Brilliant Blue R-250 (Bio-Rad, Hercules, CA, USA), while densitometric analyses were performed using a Fluor-S Max MultiImager (Bio-Rad, Hercules, CA, USA), and relative quantification of histone acetylation and histone H2Ax phosphorylation signals were normalized to the H1 signal as a control.

#### 3.2.8. Protein Analysis: SDS-Page and Western Blot

HT29 cells were seeded at a density of 5.0 × 10^4^ cells/cm^2^ in a Petri dish and treated with 50 nM of compound **4** for 48 h. Cells were then washed with PBS and incubated with the lysis buffer radio immunoprecipitation assay (RIPA) at 4 °C for 15 min with agitation. The proteins were quantified using the Bio-Rad protein assay (Bio-Rad, Hercules, CA, USA) and analyzed by SDS-PAGE and Western blot.

For the analysis of protein phosphorylation, protein samples (30 μg) were separated on 12% SDS-PAGE in Tris-glycine buffer by electrophoresis at 200 V for 1 h. The membranes were incubated overnight at 4 °C with a primary rabbit anti-phosphotyrosine antibody (Cell Signaling Technology, Danvers, MA, USA), and the bands were revealed with Chemidoc MP Imaging System (Bio-Rad) after incubation for 1 h at room temperature with peroxidase-conjugated secondary rabbit antibody (Cell Signaling Technology, Danvers, MA, USA). Densitometric analysis was performed with ImageLab 6.1 software (Bio-Rad, Hercules, CA, USA), and bands were normalized to the respective total protein content. For SCD1 analysis, protein samples were resolved on a 10% gel in MOPS buffer at 200 V for 45 min. After electrophoresis and Western blot, the nitrocellulose membrane was incubated overnight at 4 °C with primary antibody anti-SCD1 (Biorbyt, Durham, NC, USA) diluted in PBS-TWEEN 20 0.1%. The membrane was incubated for 1 h with a secondary horseradish peroxidase-conjugated antibody (GE Healthcare, Chicago, IL, USA) and then detected as described above. Quantification was completed by a Fluor-S Max MultiImager (Bio-Rad, Hercules, CA, USA) using a β-tubulin signal as a control.

#### 3.2.9. Confocal Microscopy

HT29 cells were grown on glass coverslips, treated as described above, and then washed three times with PBS. The intracellular accumulation of neutral lipids was assessed using LipidTOX™ Red Neutral Lipid Dye (Thermo Fisher Scientific, Waltham, MA, USA) according to the manufacturer’s indications. Specimens embedded in Mowiol were analyzed by confocal microscopy using a Nikon C1si confocal microscope (Nikon, Tokyo, Japan). Fluorescence was excited with a laser tuned at 595 nm, and detected at 650 nm.

#### 3.2.10. RNA Isolation and Quantitative Real-Time PCR (qRT-PCR)

Total RNA from control- and **4**-treated HT29 cells was isolated using the RNeasy Mini Kit (Qiagen, Hilden, Germany) according to the manufacturer’s protocol. RNA was quantified using the NanoDrop spectrophotometer (Thermo Fisher Scientific, Waltham, MA, USA). RNA (1 µg) was transcribed into cDNA using the PrimeScript RT Master Mix (Perfect Real Time) (Takara Bio, Kusatsu, Japan) according to the manufacturer’s instructions. Real-time PCR was performed using the Sybr Premix Ex Taq Takara kit (Takara Bio, Kusatsu, Japan) and the LightCycler 2.0 instrument (Roche Diagnostic, Basel, Switzerland). The following primers (Sigma-Aldrich, Milan, Italy) were used: 5′-CGCAGGCATCAACCCAGATT-3′ (forward) and 5′-CTGTAGCCCACGAGTGTCTC-3′ (reverse) for FASN detection, 5′-CCTAGAAGCTGAGAAACTGGTGA-3′ (forward) and 5′-ACATCATCAGCAAGCCAGGT-3′ (reverse) for SCD1 detection, and 5′-CCAACCGCGAGAAGATGA-3′ (forward) and 5′-CCAGAGGCGTACAGGGATAG-3′ (reverse) for the detection of β-actin. The first gene analyzed was the housekeeping gene β-actin, and its CT values were used as standards in the ΔΔCT method of result analysis. cDNA samples from HT29-treated cells were compared with untreated control cells.

#### 3.2.11. Statistical Analysis

Experiments were performed in triplicate and repeated at least three times. Results were averaged, and the standard deviation was calculated. To determine statistical significance, an unpaired two-tailed Student’s *t*-test was used between 2 different independent groups. A *p*-value below 0.05 was considered significant.

## 4. Conclusions

Continuing our efforts to develop hydroxystearic acid derivatives, in this work, we have constructed a series of hydroxystearic acids in which the hydroxyl group at C-9 or C-9 and C-10 has been functionalized and fully characterized as *t*-butyldimethylsilyl ethers. The strategic application of bioorganosilicon in HSAs led to the development of compounds with biological activity far superior to the original HSAs, acting at nanomolar concentrations. With the exception of compounds **8** + **9** and **10**, all others act at nanomolar concentrations in all cell lines tested, and among these, compound **4** stands out for its potency. Compound **4** is the silylated derivative of (*R*)-9-HSA, the most active free acid both as an anticancer agent [1,2,3,5,62] and as a component of PAHSAs [11]. Administration of compound **4** at 50 nM for 24 h induces an antiproliferative effect with a significant accumulation of cells in the G0/G1 phase of the cell cycle, followed by a reduction in the S phase. This effect is not mediated by the triggering of ROS but is associated with a reduction in signaling mediated by proteins with tyrosine kinase activity. Interestingly, a strong reduction in intracellular Mg concentration is observed, but this does not cause DNA damage. After 6 h of treatment, epigenetic modifications of histone proteins are highlighted, characterized by hyperacetylation of histones H2/H3 and H4. These epigenetic modifications are in turn accompanied by a significant reduction in the expression of the FASN gene, while the expression of SCD1 decreases after 24 h of treatment. These changes in gene expression lead to a different cellular phenotype characterized by a decrease in neutral lipids, suggesting that a reprogramming of lipid metabolism has taken place. It is in the epigenetic changes in histones that the important differences between silylated and free (*R*)-9-HSA become apparent. While (*R*)-9-HSA induces hyperacetylation of histone H4 [2,5], the silylated derivative also increases that of H2/H3, leading to different and stronger biological effects.

Although it remains to be clarified whether the effects induced by compound **4** are due to increased lipophilicity or other effects mediated by different targets compared to (*R*)-9-HSA, we have, nevertheless, obtained an effective compound that can not only block cell growth but also reprogram lipid metabolism in colon cancer.

## Figures and Tables

**Table 1:** In vitro cytotoxic activity of six compounds against tumor cell lines ^a^.

Compound	HT29	MCF7	HeLa	U2OS	J6
**8** + **9**	na	na	na	na	na
**10**	na	na	na	na	na
**11** + **12**	26.11 ± 0.43	na	132.5 ± 0.16	50.40 ± 0.30	na
**13**	25.32 ± 0.27	na	131.8 ± 0.21	41.95 ± 0.29	na
**3**	84.76 ± 0.10	134.0 ± 0.27	133.7 ± 0.32	29.33 ± 0.23	151.6 ± 0.31
**4**	20.31 ± 0.24	211.6 ± 0.23	166.1 ± 0.33	36.81 ± 0.18	214.1 ± 0.26

^a^ Values are reported as nM concentration and are means of four experiments. (na = not active).

**Table 2:** In vitro cytotoxic activity of **4** against colon cancer (HT29, HCT116, and Caco-2) and normal (I407) cell lines ^a^.

4	IC_50_ nM	S.I.
HT29	20.88 ± 0.21	2.96
HCT116	21.49 ± 0.16	2.88
Caco-2	23.39 ± 0.12	2.65
I407	61.91 ± 0.26	-

^a^ Values are given in nM and are means of four experiments.

## Data Availability

Data are contained within the article and Supplementary Materials.

## Supplementary Materials

The following supporting information can be downloaded at: https://www.mdpi.com/article/10.3390/molecules30030440/s1, Figures S1–S18: Copies ^1^H-NMR, ^13^C-NMR and mass spectra of novel compounds.
