# Supplementary material for: New Compounds with Enhanced Biological Activity Through the Strategic Introduction of Silylated Groups into Hydroxystearic Acids
Source: Molecules. 2025 Jan 21;30(3):440. doi: 10.3390/molecules30030440 (PMC11820896; doi:10.3390/molecules30030440)
Supplement: Supplementary file 1 [file molecules-30-00440-s001.zip › molecules-3306196-supplementary.pdf]

New Compounds with Enhanced Biological Activity Through the Strategic Introduction of Silylated Groups into Hydroxystearic Acids

Chiara Zalambani 1,†, Lorenzo Anconelli 1,†, Natalia Calonghi 1,*, Dario Telese 2, Gabriele Micheletti 2, Carla Boga 2,*, Giovanna Farruggia 1 and Eleonora Pagnotta 3

1 Department of Pharmacy and Biotechnology, University of Bologna, Via San Donato 15,

40127 Bologna, Italy; chiara.zalambani2@unibo.it (C.Z.); lorenzo.anconelli3@unibo.it (L.A.);

giovanna.farruggia@unibo.it (G.F.)

2 Department of Industrial Chemistry 'Toso Montanari', Alma Mater Studiorum Università di Bologna,

Via Piero Gobetti 85, 40129 Bologna, Italy; dariotelese@virgilio.it (D.T.);

gabriele.micheletti3@unibo.it (G.M.)

3 Research Centre for Cereal and Industrial Crops (CREA-CI), CREA Council for Agricultural Research and Economics, Via di Corticella 133, 40128 Bologna, Italy;

eleonora.pagnotta@crea.gov.it

* Correspondence: natalia.calonghi@unibo.it (N.C.); carla.boga@unibo.it (C.B.); Tel.: +39-051-2091231 (N.C.); +39-051-2093616 (C.B.)

† These authors contributed equally to this work.

Supporting information

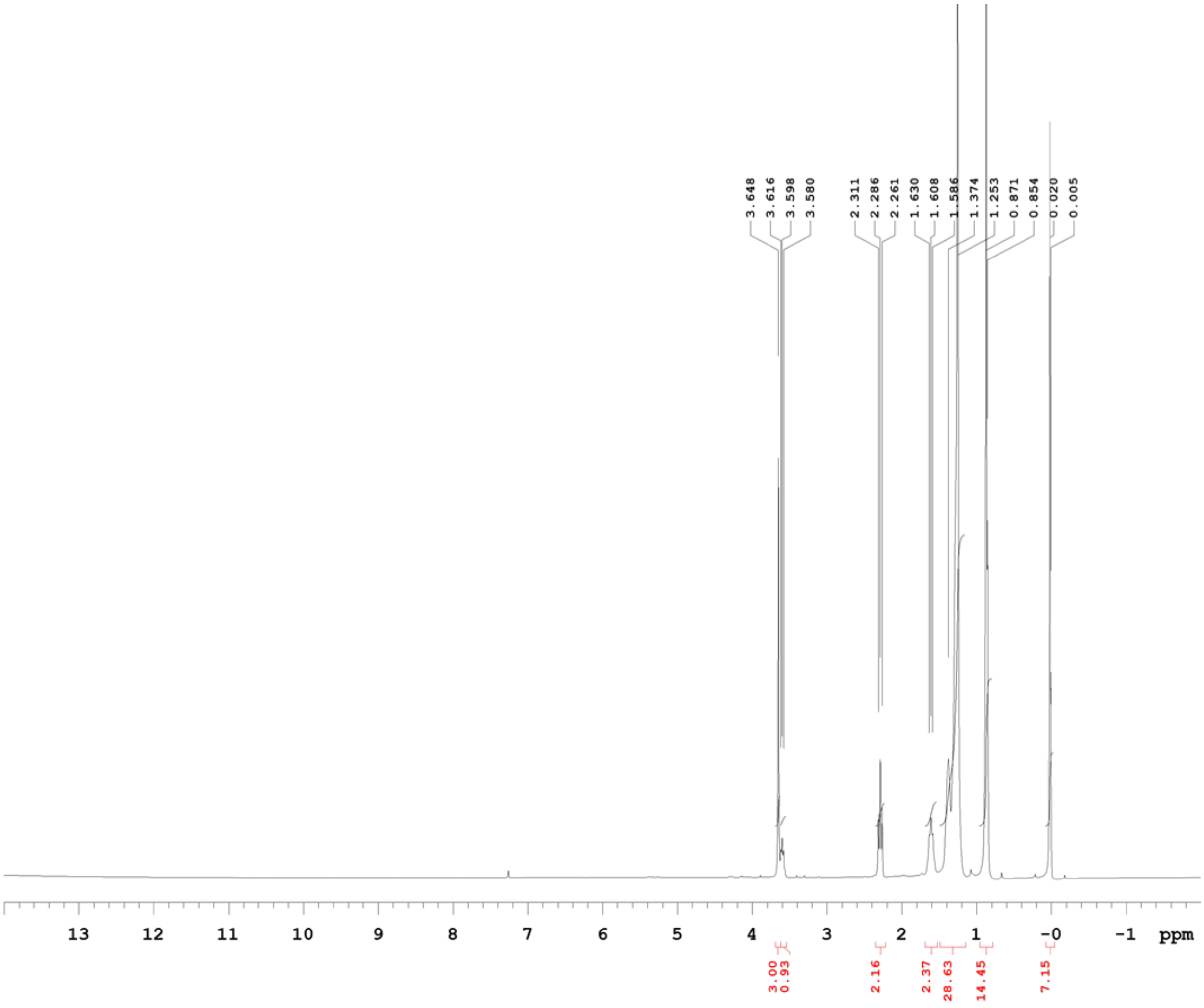

Figure S1. ^{1}HNMR spectrum ($CDCl_3$, 300 MHz) of compound **3**

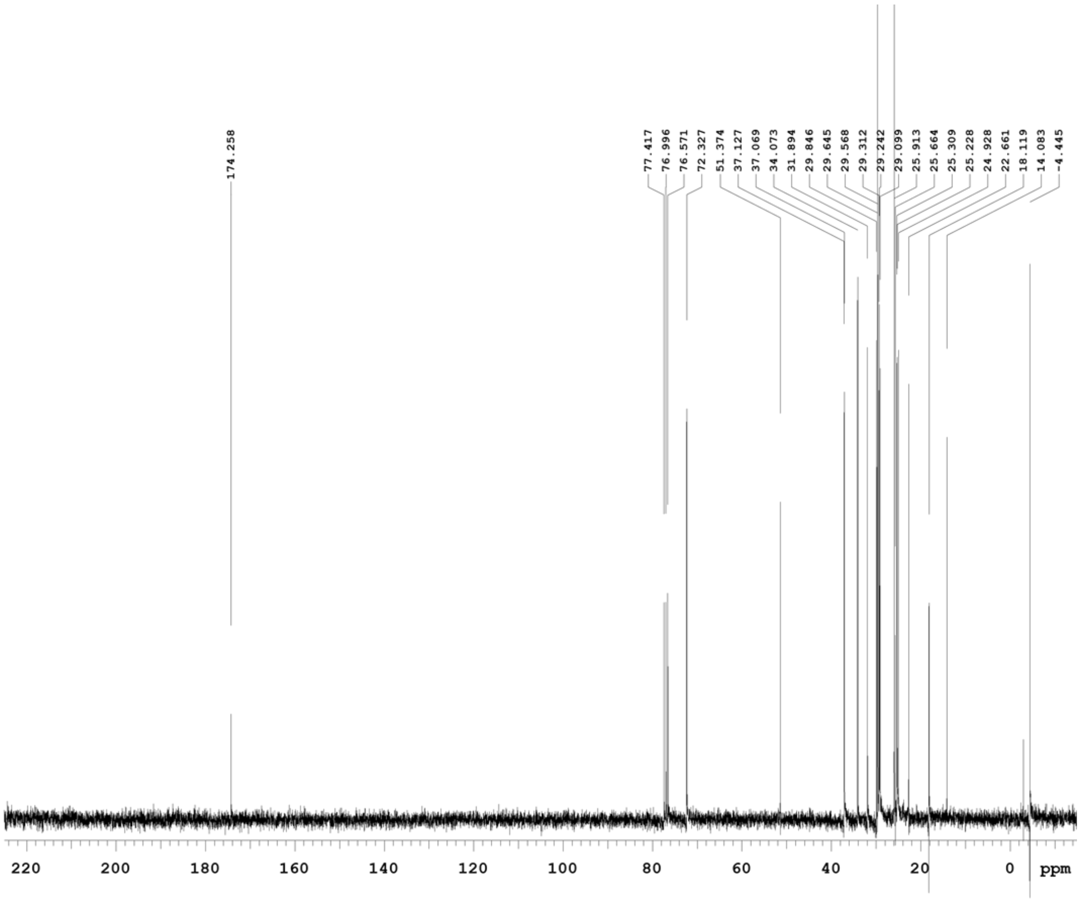

Figure S2. ^{13}CNMR spectrum ($CDCl_3$, 75 MHz) of compound **3**

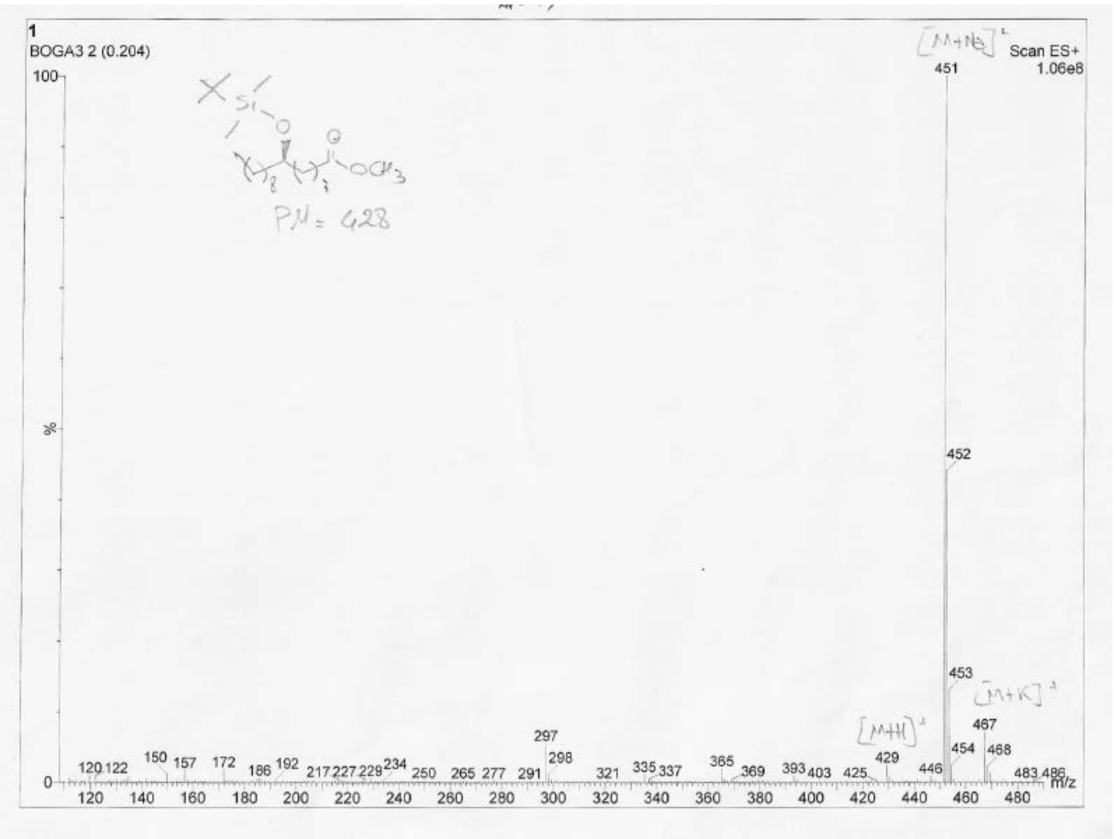

Figure S3. ESI-MS[-] spectrum of compound **3**

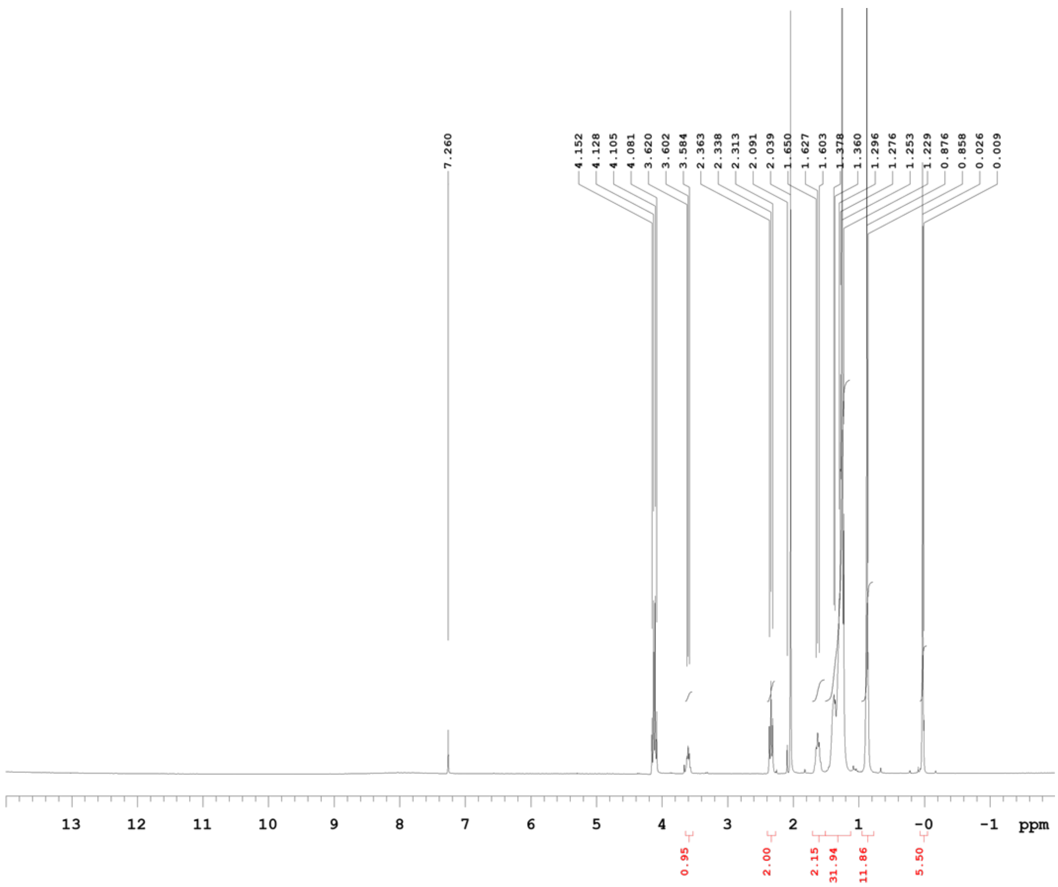

Figure S4. ^{1}HNMR spectrum ($CDCl_3$, 300 MHz) of compound **4**

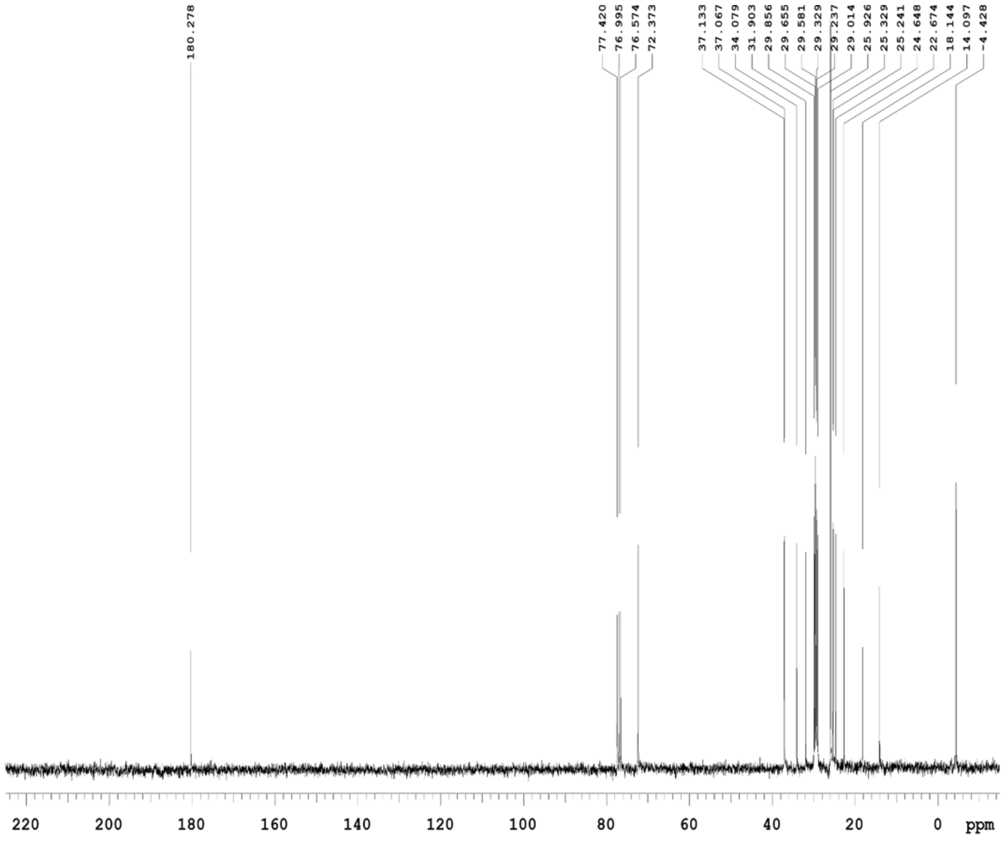

Figure S5. ^{13}CNMR spectrum ($CDCl_3$, 75 MHz) of compound **4**

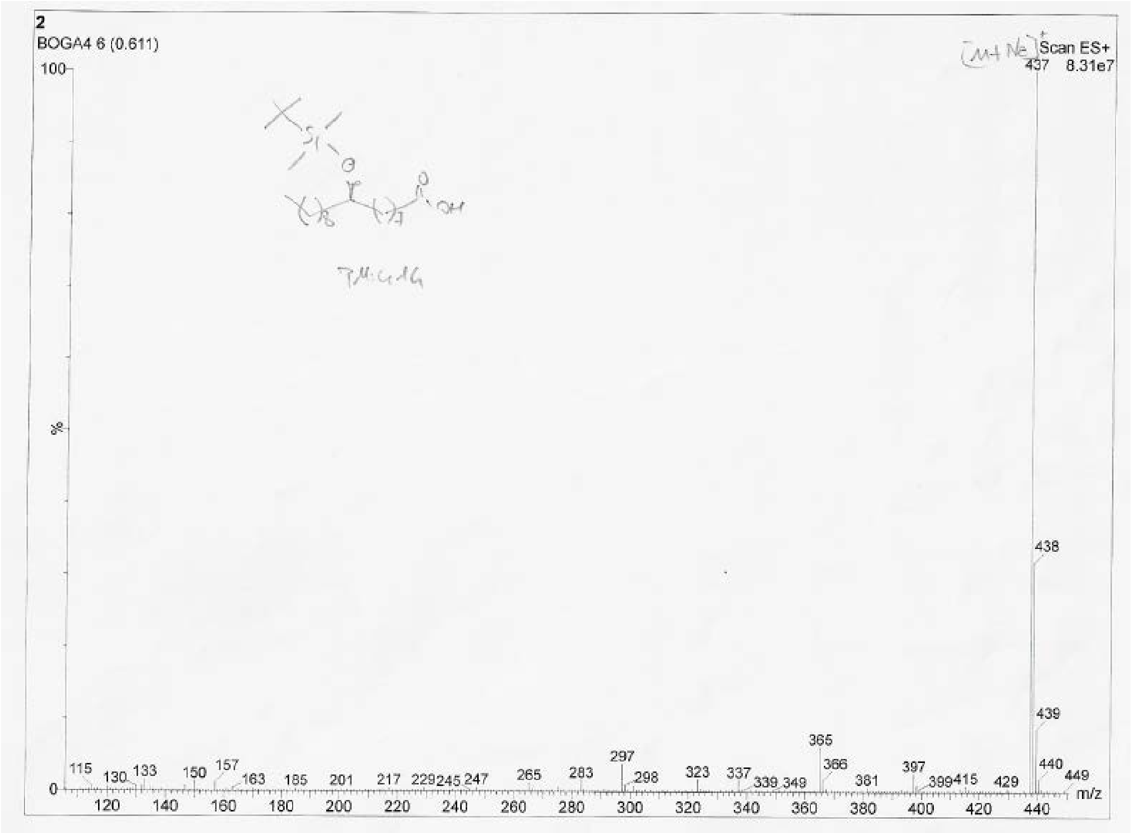

Figure S6. ESI-MS$^+$ spectrum of compound **4**

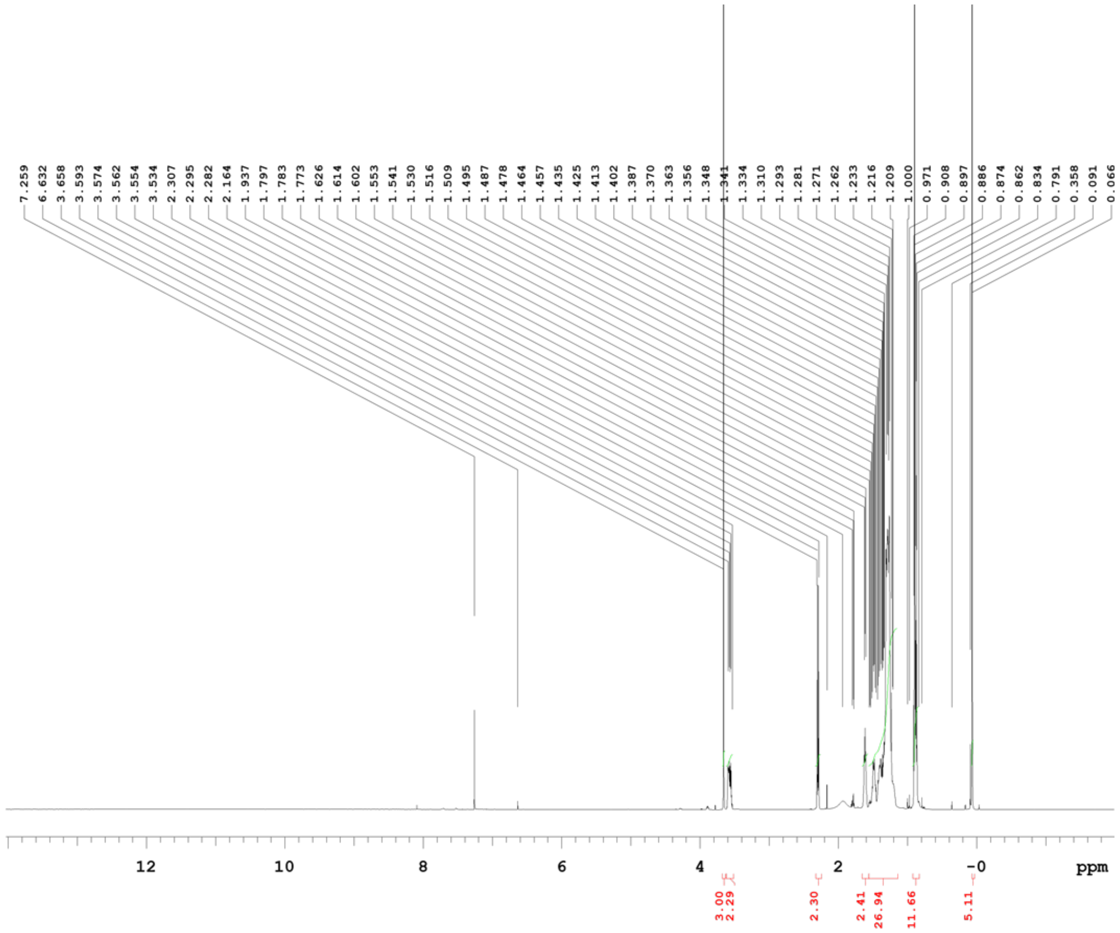

Figure S7. ^{1}HNMR spectrum ($CDCl_3$, 600 MHz) of compounds **8+9**

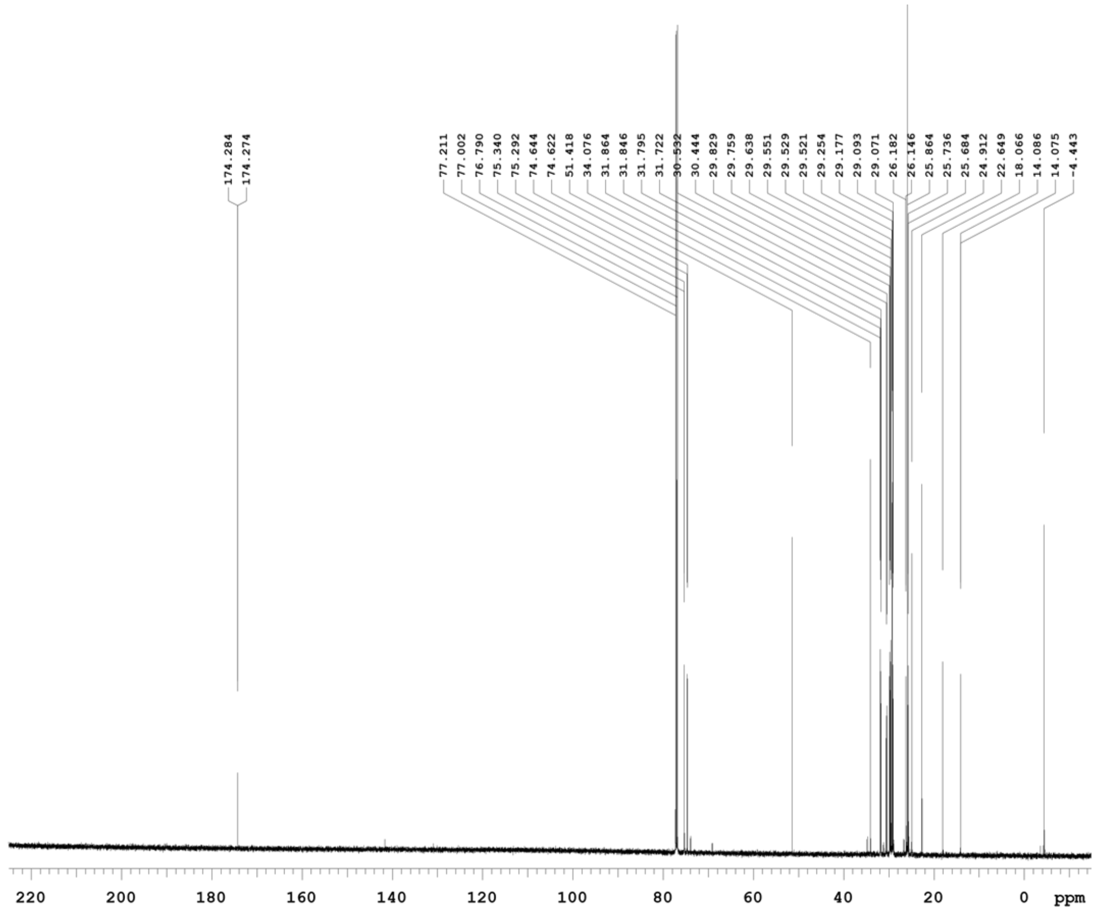

Figure S8. ^{13}CNMR spectrum ($CDCl_3$, 150 MHz) of compounds **8+9**

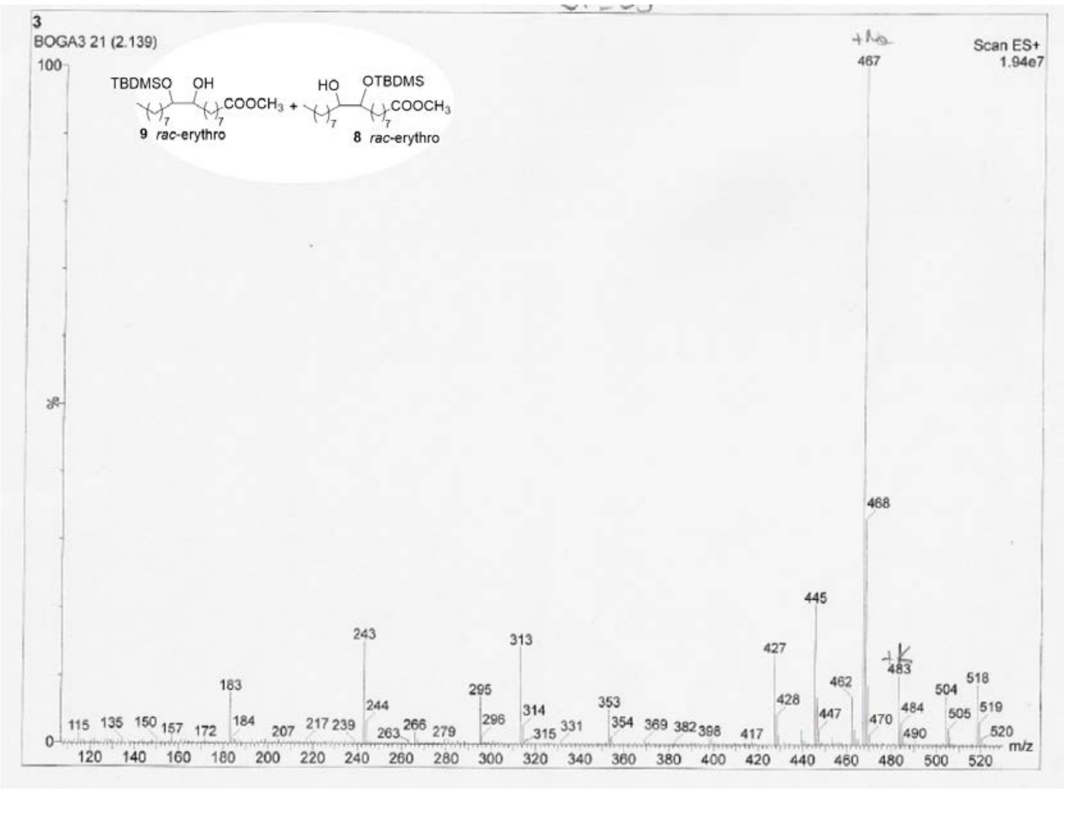

Figure S9. ESI-MS^{-} spectrum of compounds **8+9**

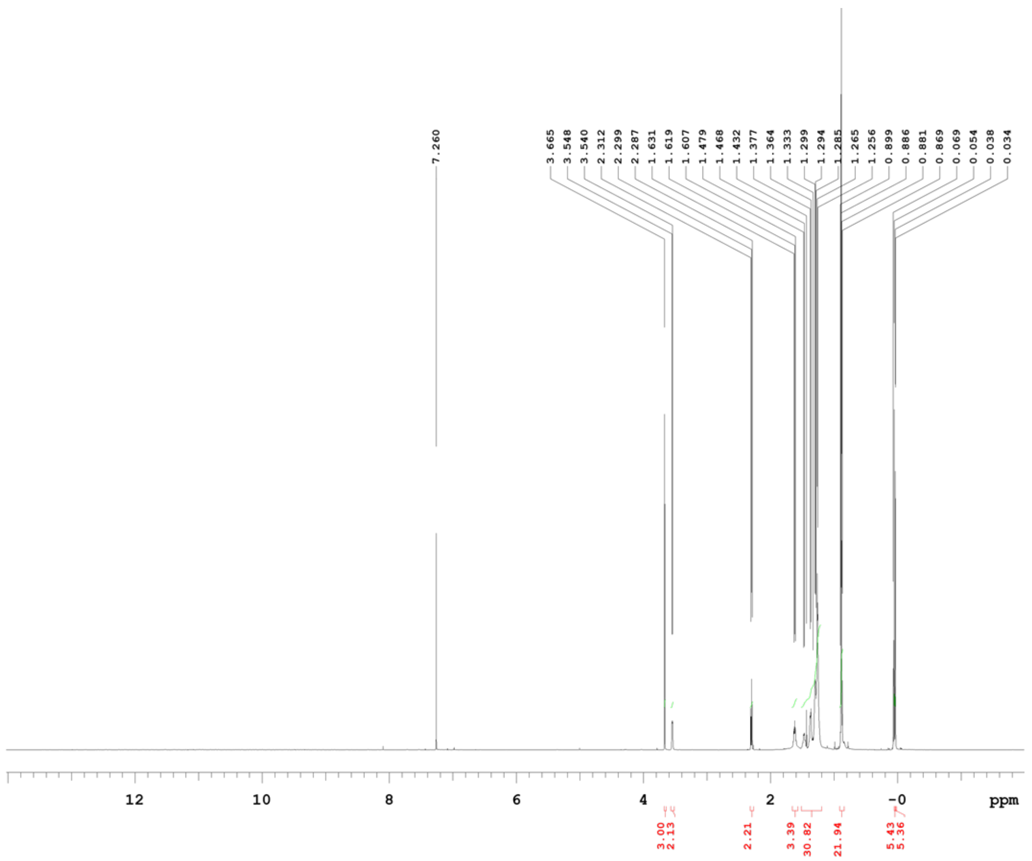

Figure S10. ^{1}HNMR spectrum ($CDCl_3$, 600 MHz) of compound **10**

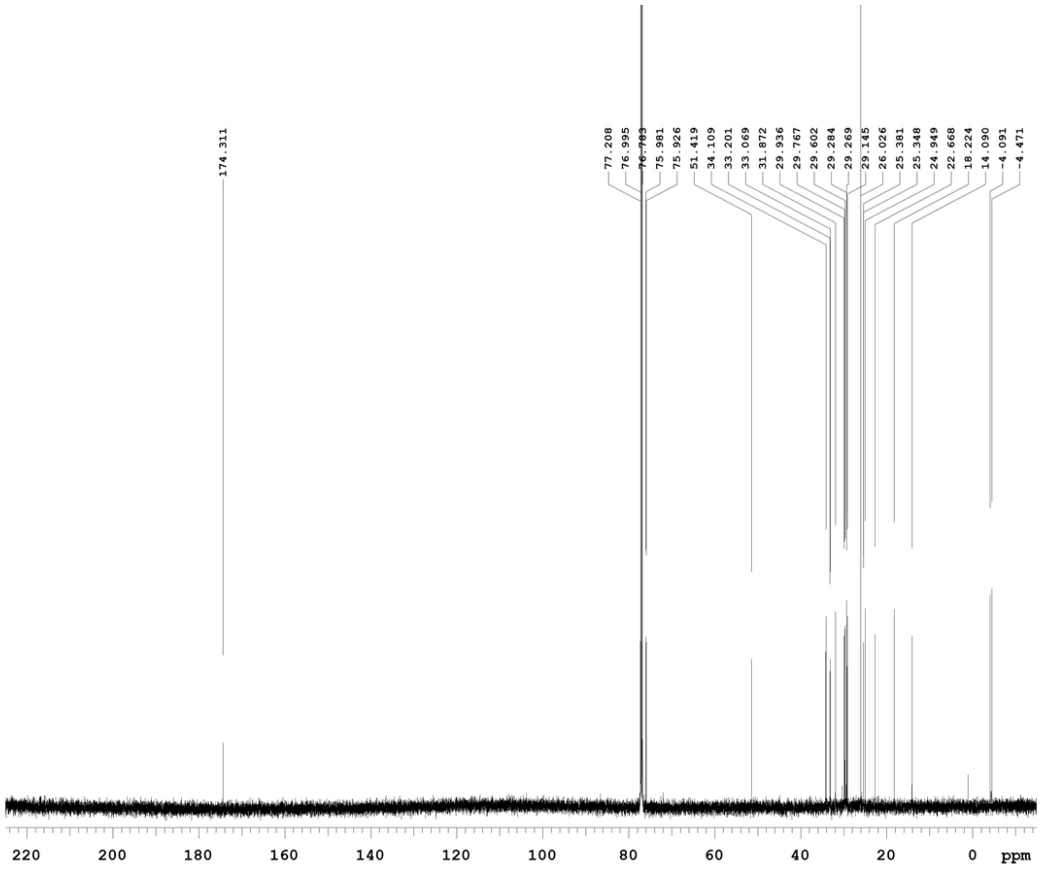

Figure S11. ^{13}CNMR spectrum ($CDCl_3$, 150 MHZ) of compound **10**

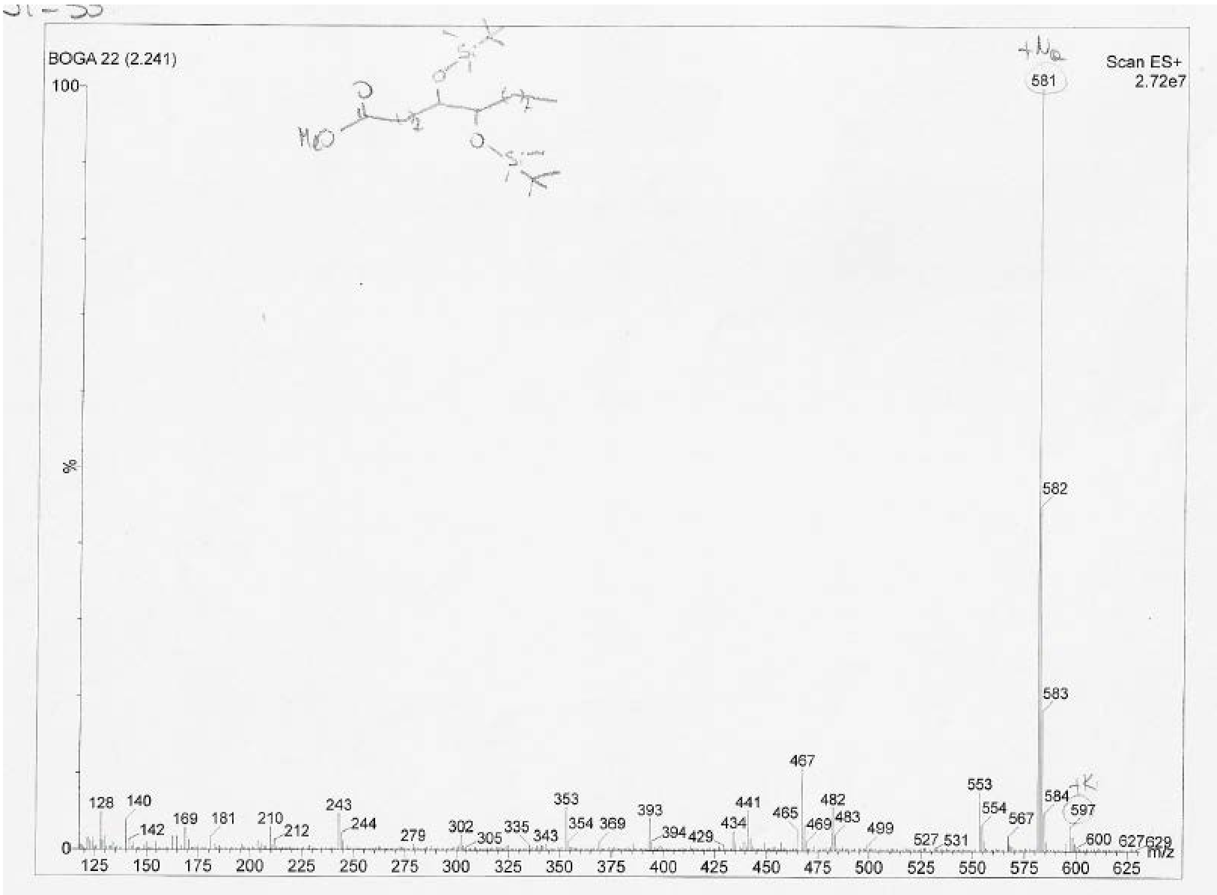

Figure S12. ESI-MS$^+$ spectrum of compound **10**

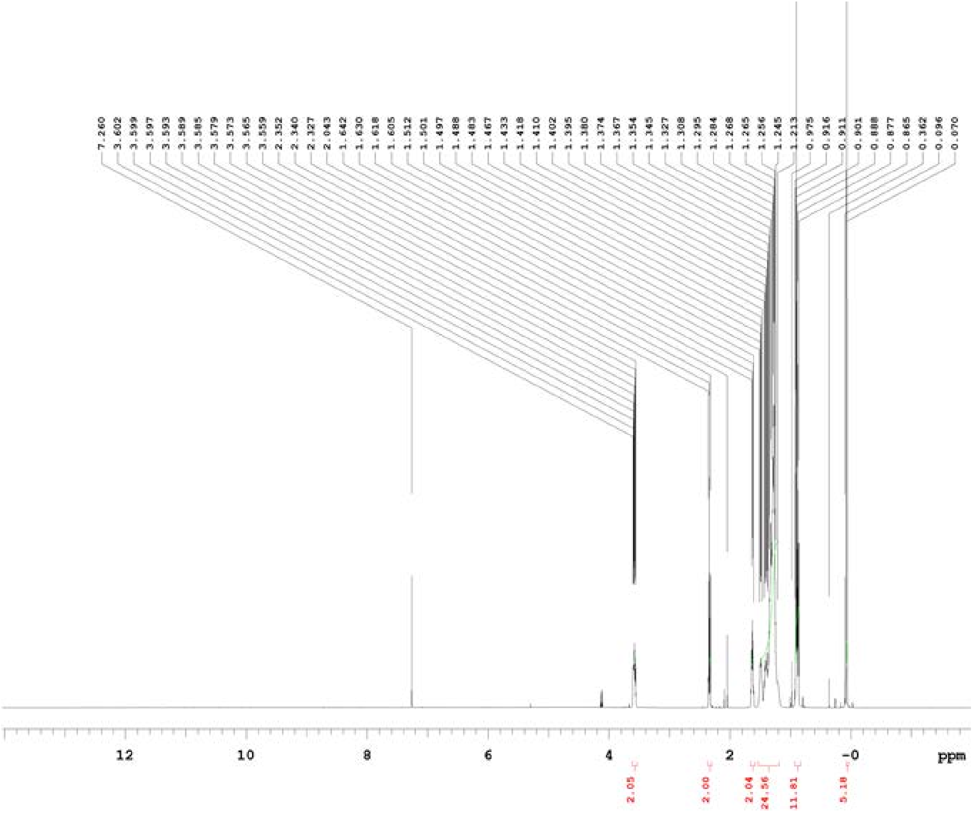

Figure S13. ^{1}HNMR spectrum ($CDCl_3$, 600 MHz) of compounds **11+12**

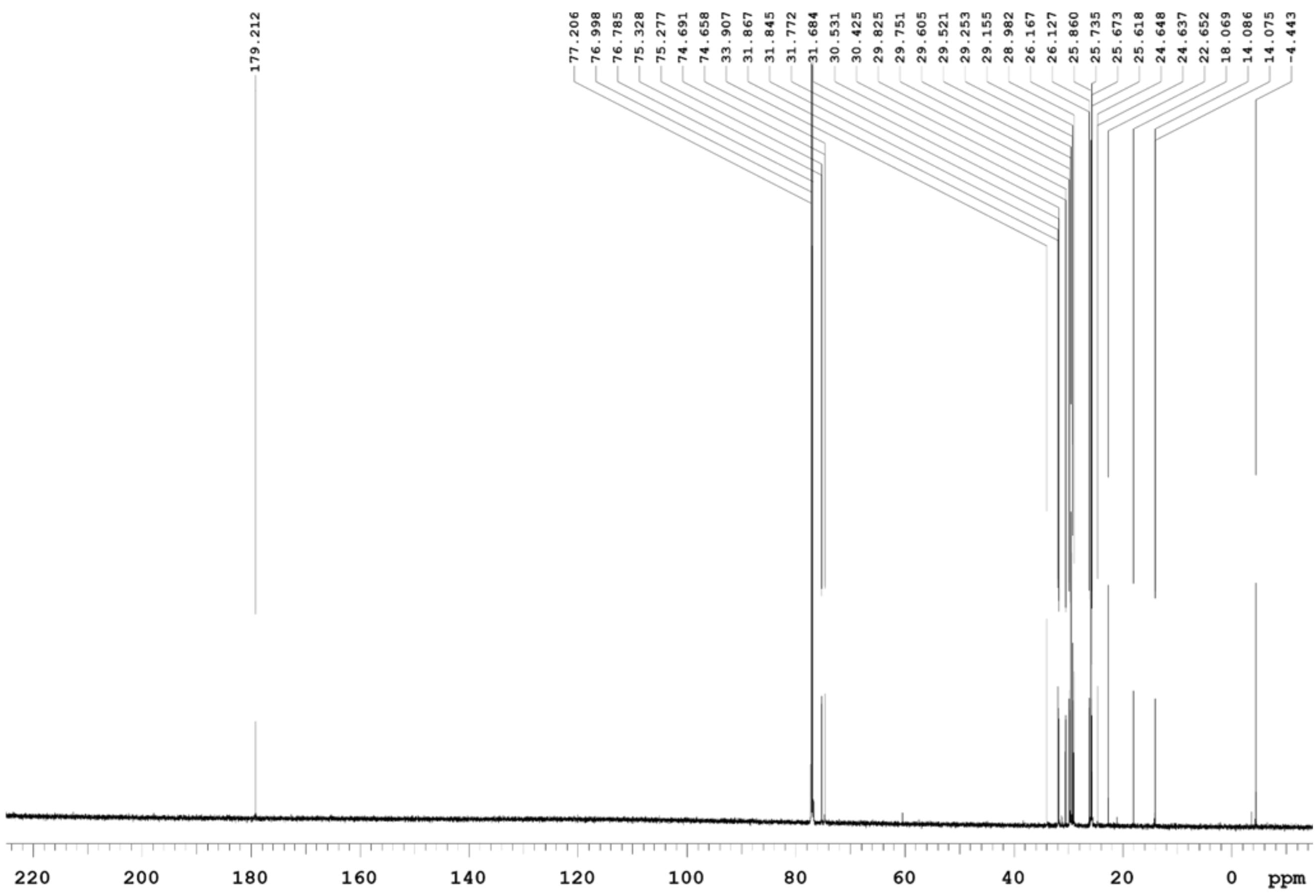

Figure S14. ^{13}CNMR spectrum ($CDCl_3$, 150 MHz) of compounds **11+12**

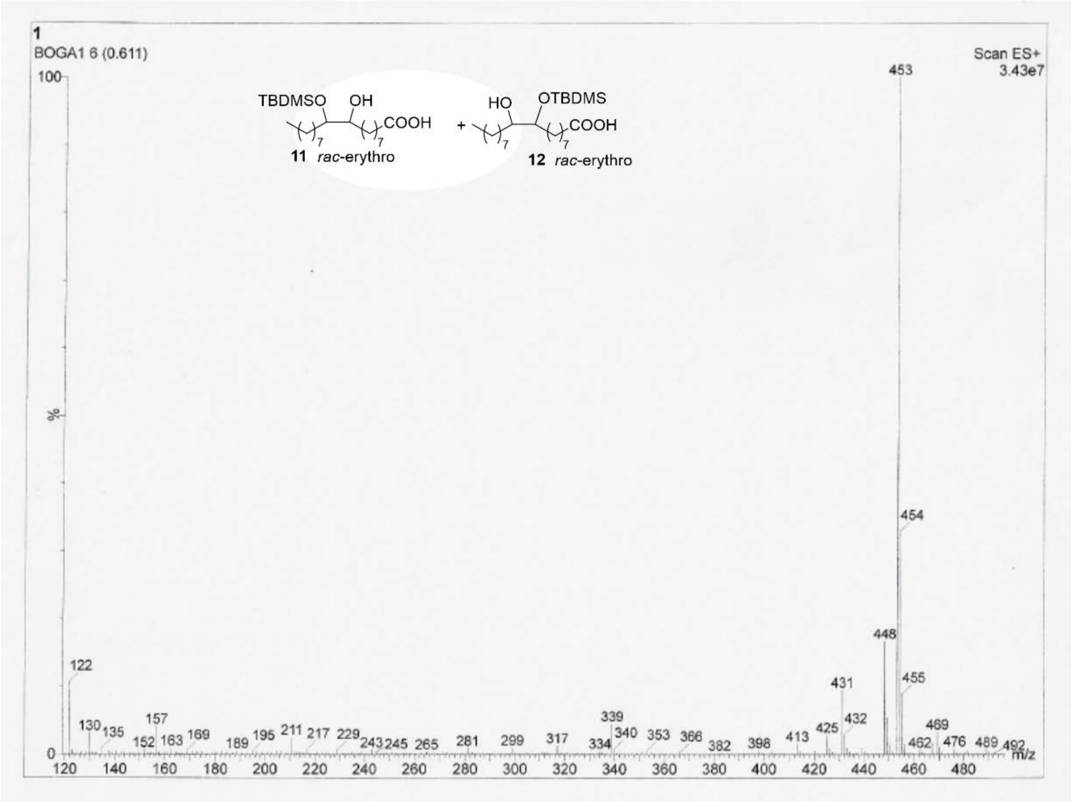

Figure S15. ESI-MS$^+$ spectrum of compounds **11+12**

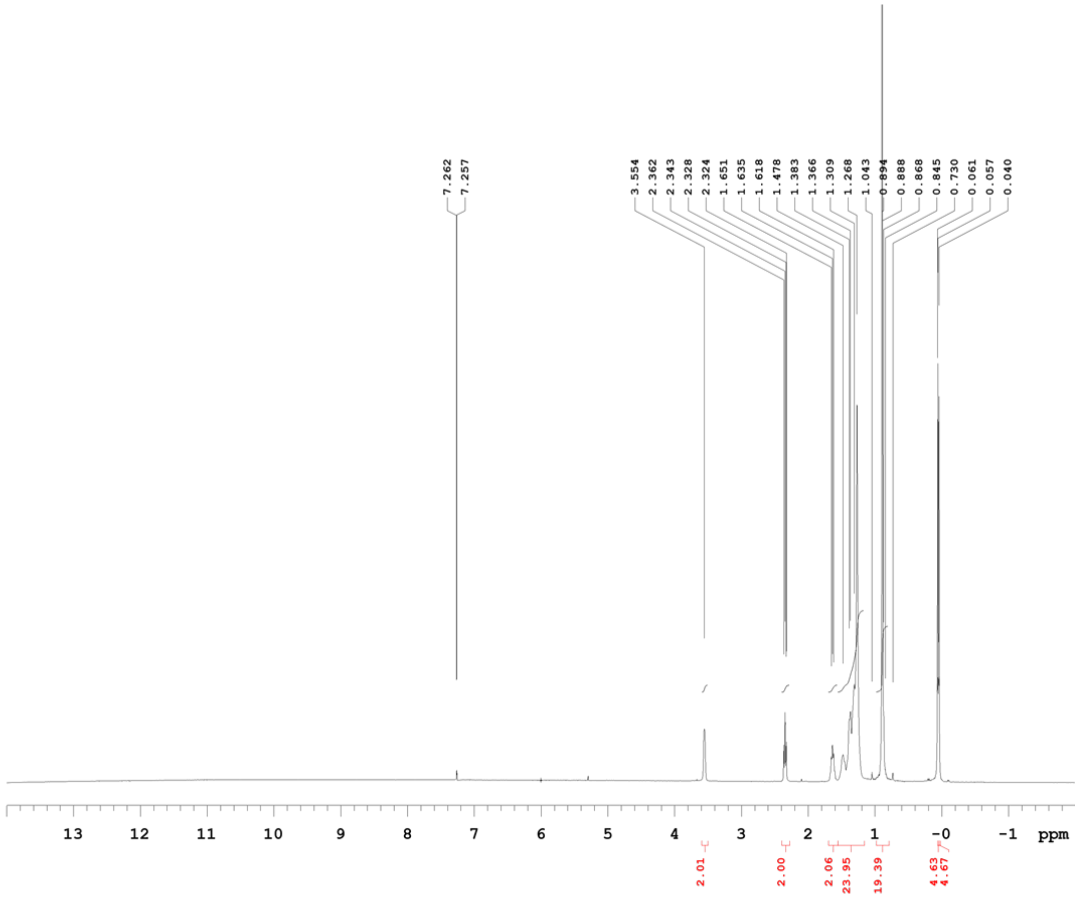

Figure S16. ^{1}HNMR spectrum ($CDCl_3$, 400 MHz) of compounds **13**

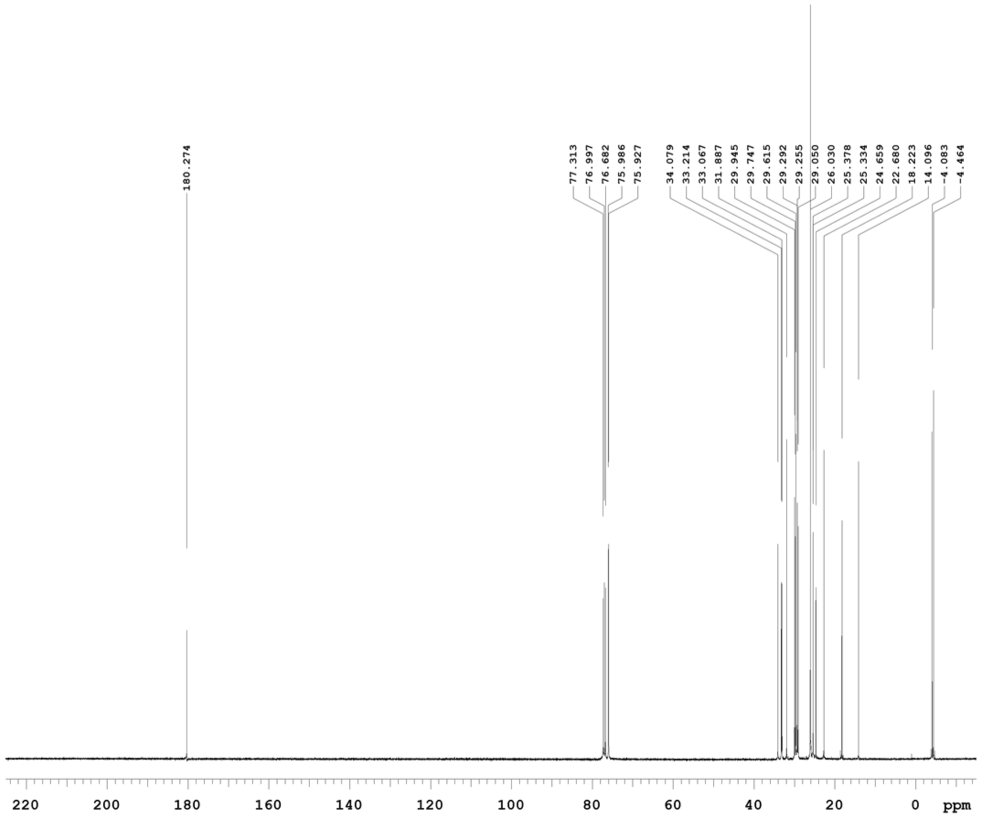

Figure S17. ^{13}CNMR spectrum ($CDCl_3$, 100 MHz) of compound **13**

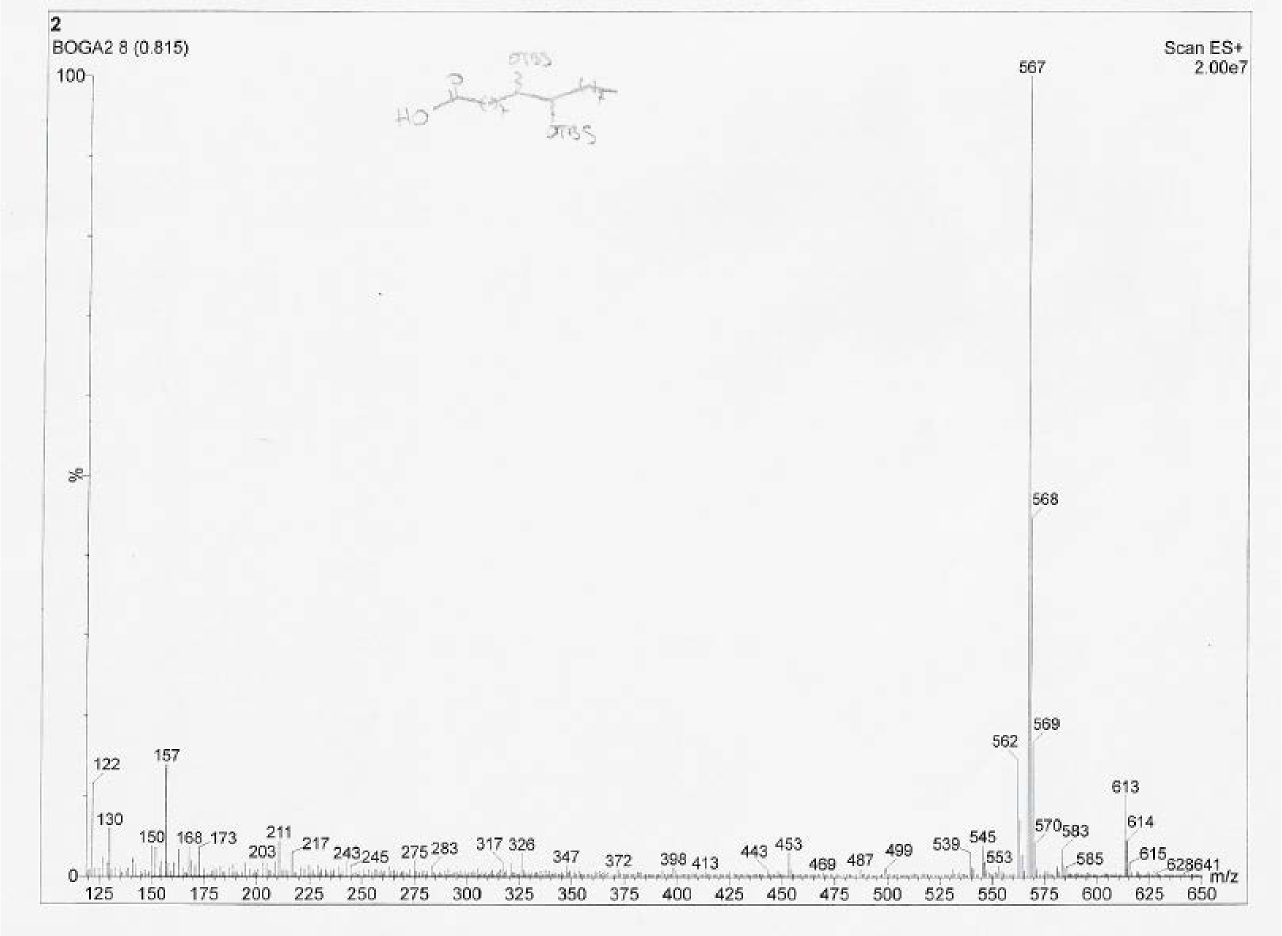

Figure S18. ESI-MS$^+$ spectrum of compound **13**
